# A Systematic Literature Review of Trauma Systems: An Operations Management Perspective

**DOI:** 10.1177/27536351241310645

**Published:** 2025-01-16

**Authors:** Zihao Wang, Bahman Rostami-Tabar, Jane Haider, Mohamed Naim, Javvad Haider

**Affiliations:** 1 Cardiff Business School, Cardiff University, Cardiff, UK; 2 Consultant in Rehabilitation Medicine, National Rehabilitation Centre, Nottingham University Hospitals NHS Trust, UK

**Keywords:** Trauma systems, operations management, literature review

## Abstract

**Background:**

Trauma systems provide comprehensive care across various settings, from prehospital services to rehabilitation, integrating clinical and social care aspects. Established in the 1970s, these systems are pivotal yet under-researched in their operational management. This study aims to fill this gap by focussing on the integration of operations management (OM) techniques to enhance the efficiency and effectiveness of trauma systems. By leveraging proven OM strategies from other healthcare sectors, we seek to improve patient outcomes and optimise system performance, addressing a crucial need for innovation in trauma care operations.

**Methodology:**

A systematic literature review was conducted using the PICOTS framework to explore operational aspects of trauma systems across varied settings, from emergency departments to specialised centres. Searches were performed in 5 databases, focussing on articles published from 2006 to 2024. Keywords related to operational research and management targeted both trauma systems and emergency management services. Our method involved identifying, synthesising, and summarising studies to evaluate operational performance, with a specific emphasis on articles that applied operational research/management techniques in trauma care. All eligible articles were critically appraised using 2 quality assessment tools.

**Results:**

Employing Donabedian’s framework to analyse the quality of trauma systems through structure, process, and outcome dimensions, our systematic review included 160 studies. Of these, 5 studies discussed the application of the Donabedian evaluation framework to trauma systems, and 14 studies examined structural elements, focussing on the location of healthcare facilities, trauma resource management, and EMS logistics. The 63 studies on process indicators primarily assessed triage procedures, with some exploring the timeliness of trauma care. Meanwhile, the 78 outcome-oriented studies predominantly evaluated mortality rates, alongside a smaller number assessing functional outcomes.

**Conclusion:**

Existing evaluation metrics primarily focussed on triage accuracy and mortality are inadequate. We propose expanding these metrics to include patient length of stay (LOS) and rehabilitation trajectory analyses. There is a critical gap in understanding patient flow management and long-term outcomes, necessitating focussed research on LOS modelling and improved rehabilitation data collection. Addressing these areas is essential for optimising trauma care and improving patient recovery outcomes.

## Introduction

A trauma system is a comprehensive, coordinated network within a specific geographical region dedicated to providing complete care to all injured individuals. Integrated closely with the local public health system, this model facilitates collaboration among commissioners, healthcare providers, public health representatives, and other stakeholders involved in trauma care. Collectively, they plan, provide, and manage treatment for people injured due to major trauma.^1^ Often referred to interchangeably as a ’trauma network,’ we use the term ’trauma system’ as an umbrella to cover all related terminologies, ensuring a unified approach in our discussion.

Since the concept of organised trauma healthcare was proposed in the 1970s, numerous countries have adopted this healthcare model to enhance the survival and recovery of trauma patients.^2,3^ Developed nations, in particular, have established comprehensive trauma systems that span from prehospital care to rehabilitation. These systems not only focus on clinical care but also integrate social aspects such as injury prevention initiatives, continuous education and training, research, quality control, planning, legal frameworks, and technological advancements, contributing to their overall effectiveness.^4^ However, the full effectiveness of trauma systems usually emerges several years after their implementation.^4,5^ As trauma systems adapt effectively, it is essential to maintain a continuous quality evaluation to ensure they adapt effectively to advances in healthcare and changing patient needs.

Thorough evaluations of trauma system effectiveness are critical for informing health policy and resource allocation decisions, which ultimately enhance patient care and system productivity. Achieving these improvements largely involves operational challenges that require the identification and implementation of more efficient ways of organising and delivering trauma care. These are central concerns of Operations Management (OM), a field dedicated to the scientific development and application of tools designed to enhance productivity and support decision-making.

The integration of OM techniques has already proven highly effective across various healthcare sectors for several reasons, such as improving efficiency,^6-9^ enhancing patient outcomes,^10^ reducing patient waiting times^11,12^ and understanding the unexpected variations in system behaviour.^13,14^ Therefore, these insights strongly advocate for expanding the application of OM techniques to trauma systems, suggesting that they could similarly revolutionise trauma care by fostering a better understanding of network dynamics and addressing prevalent system challenges.

Despite the demonstrated benefits of OM tools in various healthcare settings, there is limited research on their application within trauma systems. This study addresses this gap by examining trauma systems from an OM perspective. We explore how the analytical tool-kits developed in OM have been adapted to meet the unique challenges and decision-making processes faced by various stakeholders in trauma systems, including decision-makers, delivery organisations, patients, and medical professionals.

In this paper, we aim to consolidate recent studies on OM as applied to trauma systems. The paper is structured to achieve 2 primary objectives:

First, it addresses a research gap by focussing on the application of OM tools within trauma systems, a notably underexplored area. By synthesising existing knowledge and identifying areas that have yet to be investigated, this study contributes to a more comprehensive understanding of how OM can enhance trauma care systems. Additionally, it sets an agenda for future research in this field.

Second, the paper underscores how OM techniques can improve the efficiency of trauma systems. Adopting and modifying a framework for assessing the quality of medical care,^15^ in particular looking at how OM contributes to the quality indicators related to structures, processes, and outcomes, achieves this.

The remaining part of the paper article is structured as follows: The next section introduces the research background by providing an overview of the trauma network and trauma system. The subsequent section describes the scoping and reviewing methodology adopted and the classification criteria used for the research articles identified. Then, the content analysis of each article will be based on the classification result. Finally, the research findings and gaps will be discussed, and the conclusion and future research prospects will be presented.

## Methods

### Searching strategy

Contrary to the typical clinical viewpoint, we performed a systematic literature review of trauma networks through the lens of OM. We utilise the Population, Intervention, Comparator, Outcomes, Timing and Setting (PICOTS) framework to enhance the precision and relevance of our research question, specifically tailored to the operational aspects of trauma systems.

The review focuses on systems and networks providing hospital care to patients following major trauma (P). We examine various operations management techniques and methodologies (eg, process optimisation and resource allocation) as interventions aimed at enhancing the performance of trauma systems (I). Where available, compare the application of operations management techniques to trauma systems (C). Key outcomes include system effect and performance evaluation metrics, such as trauma system setting, patient throughput, and overall patient outcomes (O). The review considers studies irrespective of the follow-up period, focussing on the interventions’ short-term and long-term impacts (T). The settings in our review range from single centres to wider network-based systems in trauma care (S).

The basic workflow of this systematic literature review incorporated (i) identifying articles that involve evaluating operational performance in trauma systems, (ii) qualitatively synthesising their contribution to the field of trauma care planning and delivery and (iii) summarising their findings, strengths, and limitations.

**Figure 1.**
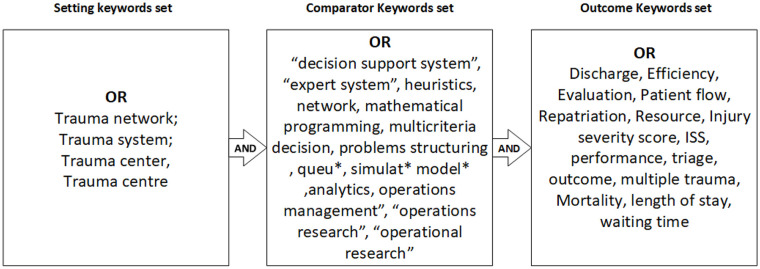
The search string. We review articles that have forecasting, inventory or stock control, and at least 1 from each of the context-specific keyword sets in their titles, abstracts or author-selected keywords.

In alignment with the outlined PICOTS framework, our search strategy employed defined keyword sets distributed across 3 aspects: ‘setting’, ‘comparator’ and ‘outcome’ (Figure 1). The ‘setting’ keywords set clearly delineate the scope of our research within various trauma care environments. The ‘comparator’ keyword set focuses on exploring operations research and management methodologies used in trauma systems. Finally, the ‘outcome’ keywords set, refining our focus to the specific clinical aspects critical for evaluating the operational interventions’ impact on trauma care. These keywords were searched across key databases, including Scopus, Web of Science, Ovid (which incorporates medical resources such as Ovid Emcare, Ovid MEDLINE^®^, Embase + Embase Classic, etc.) and Trauma Audit and Research Network (TARN) publications to ensure comprehensive coverage of multidisciplinary and healthcare-specific literature relevant to the research focus.

In addition to the above search, an additional search of the area keyword set within top-rated Q1 and Q2 journals in operational research (OR) or OM field was conducted to identify some articles applying OR/OM approaches to study trauma network systems or other similar health systems, such as emergency management service (EMS) systems. The reason for including EMS is that both trauma and emergency management service systems handle life-threatening emergencies and perform the same function in prehospital acute care. After screening and classifying these search results, 7 eligible articles were included.

**Figure 2.**
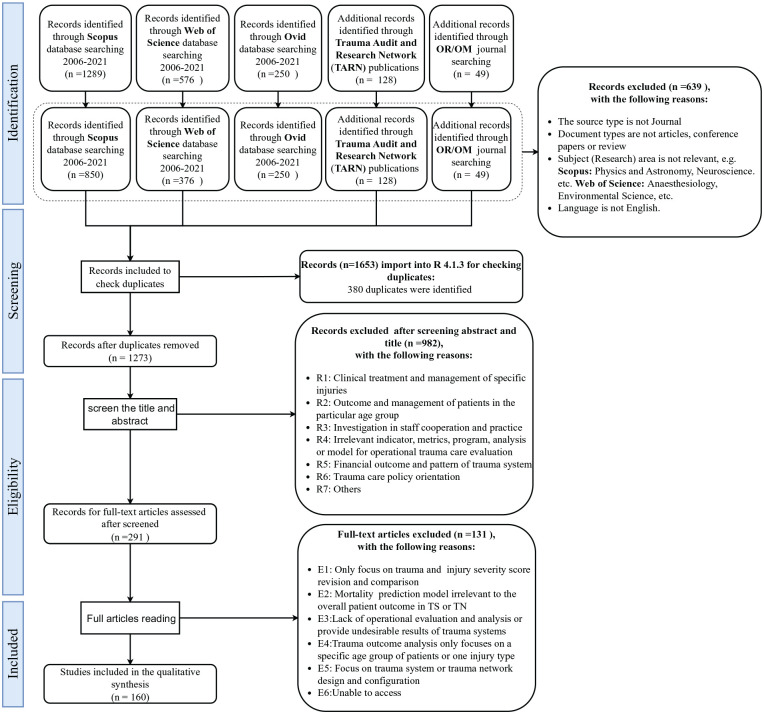
PRISMA flow chart.

This search ontology was restricted to the title, abstract, and author keywords. In addition to this, for the paper to be included in the sample, we also focussed on journal papers and searched within the English language. In terms of the searching time range, we searched papers from the last 15 years. This is mainly because most EU countries introduced trauma centres and trauma systems concepts around 2002, and they were less developed at the beginning.^16^ Therefore, there was little original research on trauma systems in Europe during that period. Besides that, no systematic review of trauma system operational performance was conducted after 2005. Finally, some irrelevant research fields (eg, physics and astronomy, neuroscience, etc.) were excluded to help focus key samples. Besides that, to gain a complete understanding of trauma network research, a certain number of valuable publications based on studies from the Trauma Audit and Research Network (TARN) were also screened. The search and review processes, along with the exclusion criteria, are summarised in the PRISMA flow diagram (Figure 2). A total of 160 papers were selected for the final review and synthesis.

### Operations management in Trauma systems: A framework

The ultimate goal of OM tools and techniques is to improve the quality of care and its related quality indicators, which are crucial to the management of a trauma system. Therefore, we use a theoretical framework proposed by Donabedian^15^ to evaluate the contribution of OM in trauma systems. We adopt this by evaluating the quality of services in 3 main dimensions, including structure, process, and outcome. This framework will facilitate the organisation of this paper and the conceptual positioning of the studies we review.

**Figure 3.**
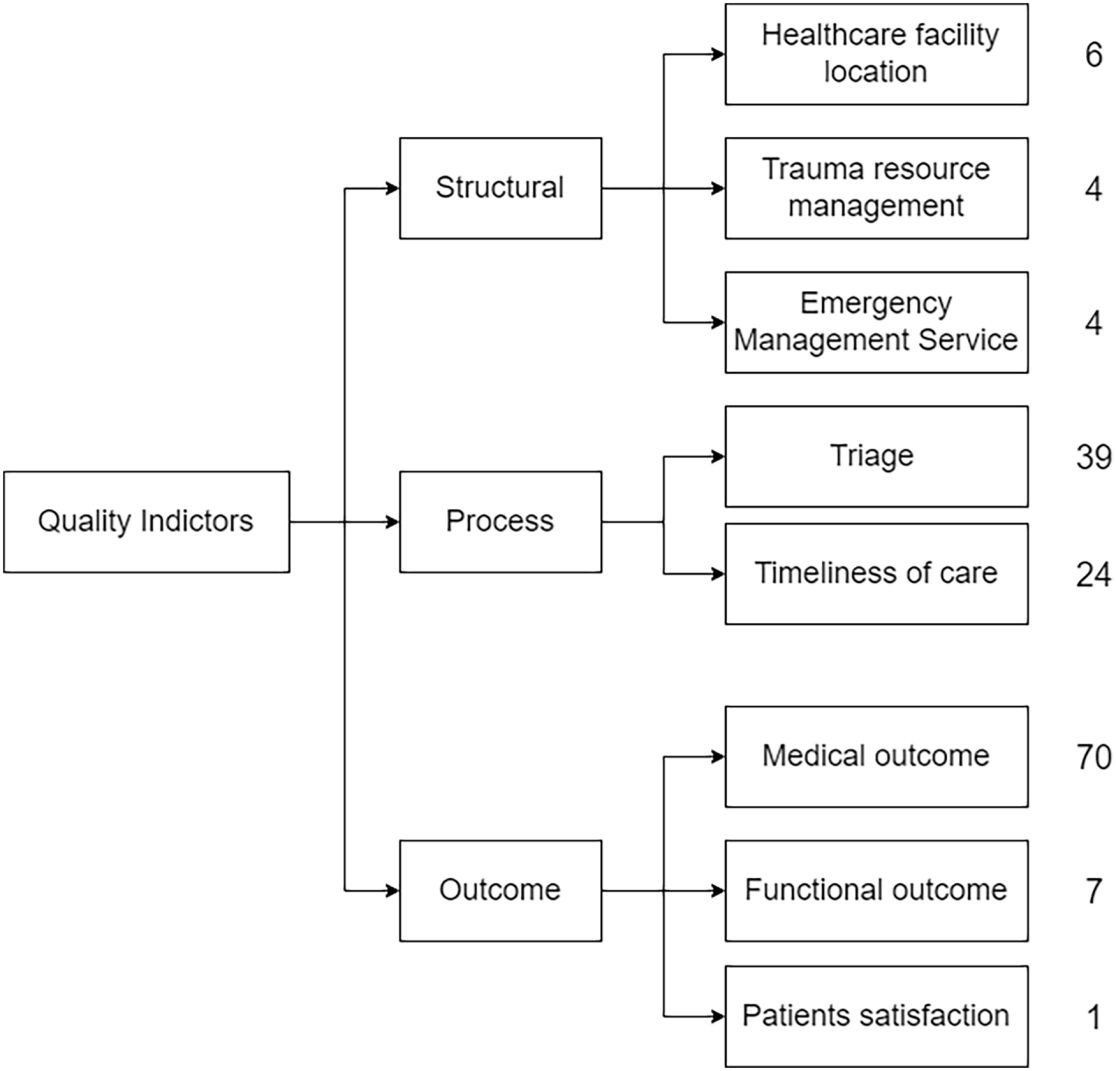
Evaluation metrics in trauma system: a theoretical framework to evaluate the contribution of operations management (a synthesis based on Donabedian^15^).

Figure 3 presents the framework that offers a three-dimensional model that is used to classify and summarise the contribution of operations management in this area. Additionally, it indicates the number of studies categorised under each dimension. These dimensions are defined as follows:

• **Structure:** Structures of health care are defined as the physical and organisational aspects of care settings (eg, facilities, equipment, personnel, operational and financial processes supporting medical care, etc.)
• **Process:** The processes of patient care sit in the middle of the diagram because they rely on the structures to provide the resources and mechanisms for participants to carry out patient care activities. In addition, processes are performed in order to improve patient health by promoting recovery, functional restoration, survival, and even patient satisfaction.
• **Outcome:** Outcome represents the patient’s condition after the treatment, including medical, function and satisfaction.

We argue that this framework is a suitable choice for our study since it effectively connects operations management tools with various quality-of-care indicators within trauma systems. Additionally, its use in multiple studies related to trauma systems underscores its relevance and applicability.^17-21^

Building upon this foundation, this paper inherently covers elements of quality management and continuous improvement within the trauma network setting in applying the Donabedian framework to assess the operational aspects of trauma systems. It is crucial to recognise that efficiently managed operations provide a foundation ensuring that resources are optimally aligned and processes are streamlined for swift response when trauma events occur. Simultaneously, quality management within this framework focuses on maintaining high standards of care during these operations, ensuring that the interventions not only occur efficiently but also lead to the best possible patient outcomes. Thus, while OM facilitates the effective arrangement and utilisation of healthcare resources, quality management and continuous improvement drive ongoing enhancements in these operations, focussing on patient health, recovery, and satisfaction as critical indicators of success.

### Quality assessment

Considering the current review includes research with multiple study types, the Mixed Methods Appraisal Tool (MMAT), a distinctive tool for appraising the quality of various study designs,^22^ was used to assess the quality of the eligible studies. Following its development,^23^ the MMAT has been validated^24,25^ and refined,^22^ proving effective for evaluating qualitative, quantitative, and mixed-method studies in mixed studies reviews. The methodological quality of each eligible paper was evaluated based on the relevant criteria outlined in the MMAT. Comments on study methods, operational orientation, and classification according to Donabedian’s framework dimensions were documented for each paper (Table 2 in Supplemental Materials). However, owing to the constraints of MMAT in assessing systematic literature reviews,^22^ the Critical Appraisal Skills Programme (CASP) checklists for systematic review and meta-analysis checklist^26^ were utilised for the assessment of 6 identified systematic review articles in this review (Table 3 in Supplemental Materials).

In the following sections, we provide the study characteristics, synthesis of the literature, and research gaps identified for the application of OM in the structure, process, and outcome dimensions of the framework in Figure 3.

## Study Characteristics

**Figure 4.**
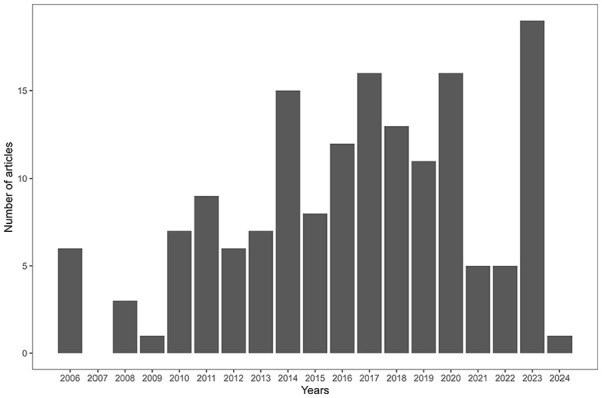
Number of publications over time.

**Figure 5.**
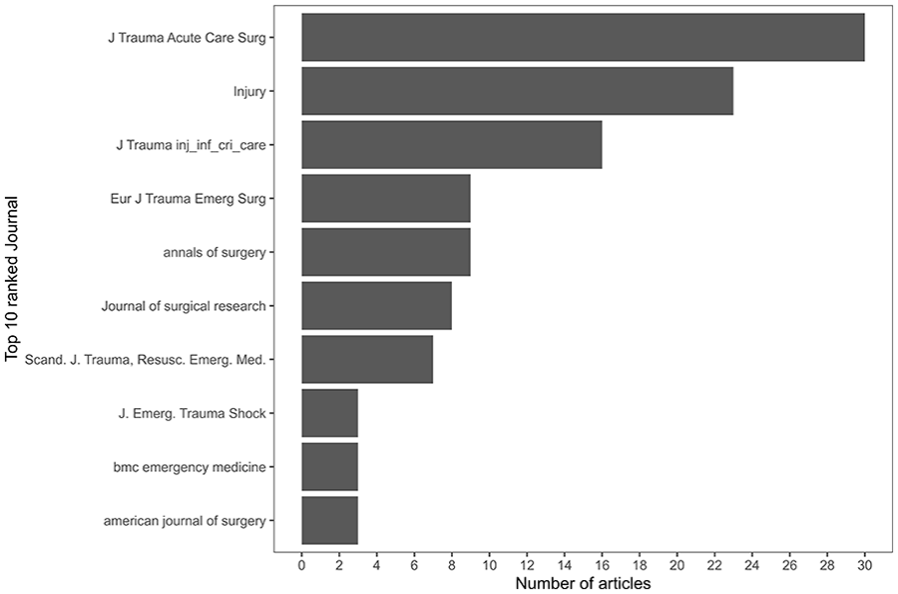
Articles published per academic journal.

Figure 4 indicates the number of articles published between 2006 and 2024, highlighting an overall increasing trend. The number of publications in 2024 does not cover the entire year (at the time of the literature search). Figure 5 presents a breakdown of the top 10 journals where the most frequently cited articles are published. The majority of these articles are found in journals that specialise in the medical domain rather than in journals dedicated to OM.

The detailed characteristics of the eligible papers are provided in Table 4 in the Supplemental Materials. It covers the study’s focus, classification of the Donabedian framework dimensions, sample size, geographical location of the trauma systems studied and the scale of the research. Most studies predominantly examined localised settings of trauma systems through 55 regional and 44 single-centre studies. Meanwhile, additional studies in cross-regional, national, and worldwide areas further enhance a comprehensive understanding of the comparison of trauma system performance. Besides that, Outcome indicators dominate the evaluation metrics featured in 78 studies, emphasising their critical role in trauma care evaluation. The majority of the studies feature smaller sample sizes, which is reflective of focussed, in-depth investigations, even though a significant portion examines extensive populations over varied time scales, here with a notable emphasis on long-term impacts and outcomes.

## Structure

Table 1 shows a summary of 14 studies focussed on the structural quality indicators of trauma systems. These studies are categorised into 3 main areas: 6 articles analyse location problems of healthcare facilities within trauma systems; 4 address trauma resource management; and another 4 explore issues related to Emergency Management Services (EMS). All studies share an operational focus and contribute to a comprehensive understanding of how the structural aspects influence trauma systems’ overall functionality and responsiveness.

**Table 1.** Overview of studies on structural aspects of trauma systems evaluation.

Reference	Specific research area	Study emphasis	General study design	Analytic methods
Cho et al^27^	Locations problems of healthcare facility	Operational	Simulation study	Mixed-Integer Nonlinear Programming (MINLP), Shifting Quadratic Envelopes Algorithm
Ahmadi-Javid et al^28^	Locations problems of healthcare facility	Operational	Survey and literature review	Comprehensive classification of healthcare facilities and reviews of relevant mathematical models and solution techniques
Bélanger et al^29^	Locations problems of healthcare facility	Operational	Simulation-optimisation study	Recursive simulation-optimisation framework combined with a discrete event simulation
Hirpara et al^30^	Locations problems of healthcare facility	Operational	Optimisation study	Nested multi-level, multi-criteria optimisation model, bi-objective optimisation for equity and effectiveness
Beck et al^31^	Locations problems of healthcare facility	Operational	Optimisation study	Geospatial analysis, Mixed integer linear programming, retrospective data analysis
Parikh et al^32^	Locations problems of healthcare facility	Operational	Optimisation study	Performance-based assessment model (PBATS), optimisation techniques, data-driven trauma network analysis
Kunene and Weistroffer^33^	Trauma resource management	Operational	Decision and data mining analysis	Multicriteria Decision Analysis (MCDA), Decision rules, Analytic Hierarchy Process (AHP), Data mining Techniques
Hyer et al^34^	Trauma resource management	Operational	Case study	Process design and focus-based restructuring
Anderson et al^36^	Trauma resource management	Operational	Optimisation study	Continuous-time Markov chain, Integer Linear Programming, Heuristic optimisation
Faraj and Xiao^35^	Trauma resource management	Operational	Case study	Expertise coordination, dialogic coordination, practice-based analysis of coordination in trauma centres
Aringhieri et al^37^	Emergency management service	Operational	Literature review	Comprehensive review, classification based on equity and uncertainty, discussion of mathematical models and solution techniques
Allon et al^39^	Emergency management service	Operational	Capacity impact analysis	Queueing Theory, Sample Selection Model, Diffusion and Fluid Approximations
Webb and Mills^38^	Emergency management service	Operational	Operational policy study	Decision modelling, economic analysis, policy incentives analysis
McHenry and Smith^40^	Emergency management service	Operational	Retrospective cohort study	Geospatial modelling, logistic regression, linear regression

### Locations problems of healthcare facility

The optimisation of trauma system locations has significantly advanced through seminal studies. Cho et al^27^ marked a pivotal enhancement in location optimisation for trauma centres and helicopter bases, achieving up to 20% improvements in benchmarks. This laid the foundation for Ahmadi-Javid et al,^28^ that proposed an integrated framework to maximise coverage efficiency, notably positioning helicopters primarily as support to ambulances and shifting the operational paradigm.

Building on these foundations, Bélanger et al^29^ introduced a decision model that synergies ambulance location with dispatch strategies through a simulation-optimisation framework, demonstrating substantial improvements in EMS management. Advancing this further, Hirpara et al^30^ developed the Nested Trauma Network Design Problem (NTNDP), optimising the distribution of Major and Intermediate Trauma Centres (MTCs and ITCs) and emphasising equity and effectiveness, particularly in improving access in rural settings.

Expanding on this groundwork, Beck et al^31^ employed geospatial and mathematical models to enhance the distribution and configuration of trauma centres, showcasing the effectiveness of data-driven approaches in optimising access to care. Parikh et al^32^ introduced a mathematical programming approach that refined the distribution and number of trauma centres based on specific performance metrics, adding a crucial dimension to trauma resource management.

Together, these studies represent a continuum of innovation in trauma system location optimisation, each building upon the last to refine strategies and methodologies. This body of work not only highlights the ongoing evolution of location management within trauma networks but also sets a promising direction for future inquiry.

### Trauma resource management

Research in trauma resource management has highlighted various strategies for enhancing care, ranging from data management to operational optimisation and the strategic placement of trauma centres. Kunene and Weistroffer^33^ demonstrate the effectiveness of multi-criteria decision analysis in managing large datasets for traumatic brain injury, effectively aligning data analysis with strategic healthcare objectives. Similarly, Hyer et al^34^ illustrate how principles derived from manufacturing can improve both clinical outcomes and economic efficiency in trauma units, suggesting that focussed operational practices can yield substantial financial and clinical benefits without adversely affecting mortality.

The critical role of coordination within trauma centres is underscored by Faraj and Xiao,^35^ who advocated for expert and dialogic coordination practices to ensure timely and error-free care. This is complemented by the findings of Anderson et al,^36^ who reported variability in the quality of trauma care depending on the time of patient arrival, with discrepancies during off-hours often due to differences in resource availability rather than patient characteristics.

Collectively, these studies underscored the importance of a holistic resource management approach in trauma networks; they emphasised the critical need for sophisticated data analysis, targeted operational improvements, and strategic coordination within trauma centres. Utilising advanced analytics and strategic planning, including geospatial analysis and performance-based assessments, is crucial for enhancing access and improving the quality of trauma care.

### Emergency management service

EMS operations are crucial in addressing a wide range of challenges within the emergency care continuum. Aringhieri et al^37^ provided a comprehensive review of Emergency Care Pathway (ECP) evaluation metrics. Their work covered a broad spectrum, including ambulance location and relocation models, dispatching and routeing policies, integration with national health systems, demand forecasting, and workload predictions, laying a strong foundation for further analytical advancements in the field. Furthermore, Webb and Mills^38^ explored the impact of economic incentives on pre-hospital triage, demonstrating how well-designed incentive structures can mitigate healthcare costs and alleviate emergency department overcrowding by effectively addressing over-triage issues. Allon et al^39^ delve into the operational dynamics of ambulance diversion, using queueing theory to assess how operational parameters affect ED and inpatient flow. Their findings reveal that the impact of ambulance diversion varies significantly based on hospital characteristics, emphasising the need for tailored strategies in different hospital settings. McHenry and Smith^40^ examined how geospatial and temporal factors influence EMS responses to major trauma. Their study highlighted the critical importance of strategic placement and refined dispatch criteria for emergency services to ensure equitable access to trauma care, especially in geographically isolated areas. The authors suggested that positioning pre-hospital critical care closer to major trauma centres could significantly enhance response efficacy.

Collectively, these studies shed light on the complex operational challenges within EMS systems. They offer a spectrum of solutions ranging from advanced system modelling to strategic resource allocation, thereby enriching both theoretical frameworks and practical interventions in trauma network operations. This body of work not only advances our understanding of EMS logistics but also sets the stage for ongoing improvements in emergency medical response systems.

## Process

Adopting Donabedian’s model for trauma care evaluation, as elucidated by Moore et al,^20^ the ‘process’ component includes the entirety of clinical and administrative procedures encountered by patients throughout their journey within a trauma network. This continuum begins with initial triage during the pre-hospital stage and extends through a wide array of in-hospital clinical interventions that directly impact a patient’s length of stay, including medical evaluations, diagnostic imaging, surgical procedures, and admission, culminating in their eventual discharge.

### Triage

Trauma triage, which is essential for the operation of a trauma network, ensures the optimal utilisation of resources and facilities by guiding the right patients to the appropriate trauma centres in a timely manner. This critical operation hinges on a robust pre-hospital infrastructure, inclusive of an effective dispatch and a well-resourced ambulance service.^41^ Embodying a 2-stage process, triage spans both field (pre-hospital) and secondary (in-hospital) assessments. Numerous studies have delved into the efficacy and methodologies of triage, focussing on field triage protocols, overall performance metrics, and the identification of key predictors for triage ‘decision-making’. All triage-related articles are summarised in Table 5 of the Supplemental Material.

#### Evaluation of field triage protocol

In terms of field triage protocol, diverse interpretations and applications can result in undertriage, leading to potential increases in mortality by failing to deliver severely injured patients to higher-level trauma centres promptly, or overtriage, which strains resources through the unnecessary allocation of minor injury cases to these centres.^42,43^ The American College of Surgeons Committee on Trauma (ACS-COT) has established guidelines featuring a 4-step algorithm with 24 criteria aimed at minimising these risks by ensuring critically injured patients are rapidly transferred to major trauma centres. These guidelines suggest a benchmark undertriage rate of 5% and an overtriage rate of up to 50% as indicators of an inclusive trauma system’s effectiveness.^44,45^ Nevertheless, achieving and maintaining these benchmarks is formidable, as highlighted by the hypothesis that efforts to decrease undertriage inadvertently inflate overtriage rates, reflecting an inversely proportional relationship between these critical metrics. This complex balance underscores the need for precise definitions and continuous refinement of triage accuracy, overtriage, and undertriage concepts to align with evolving clinical insights and operational objectives within trauma care systems.

Studies evaluating triage protocols have revealed a spectrum of approaches to improve decision-making at the trauma scene. From the early application of the T-RTS scores within the Dutch trauma system^46,47^ to more recent attempts at refining triage criteria to include less obvious yet predictive indicators,^48^ the quest for enhanced triage accuracy continues. Notably, the subjective judgement of EMS providers often prevails over strict adherence to algorithmic criteria,^44^ highlighting the need for protocols that accommodate frontline realities.

Comparative analyses of triage protocols, such as those by Shawhan et al^49^ and Follin et al,^50^ have demonstrated varying degrees of success in addressing overtriage and mistriage through the simplification of criteria or the development of novel algorithms. Magnone et al^51^ evaluated the effectiveness of a regional Italian trauma system’s field triage protocol by investigating mechanism-based trauma team activation, revealing significant protocol limitations with an 83.2% overtriage rate and identifying specific injury mechanisms that could serve as more reliable triage criteria, highlighting the need for protocol refinement. Meanwhile, Braken et al^52^ and Haider et al^53^ explored criterion adjustments to strike a more effective balance between undertriage and overtriage rates. Contrary to the pursuit of the ACS-COT’s undertriage threshold,^54^ argued for a more nuanced approach that considers the broader impact on patient outcomes beyond mortality rates.

Despite these advancements, the collective body of research, as summarised by Morris et al,^55^ indicates a persisting need for a universally recognised, simple, and generalised triage tool. Such a tool should integrate all stages of triage to form a comprehensive decision model, guiding EMS providers towards more accurate and effective patient triage in trauma care systems.

#### Triage performance and impact

Evaluating the performance of triage protocols uncovers a complex landscape where the delicate balance between undertriage and overtriage rates is pivotal. A nationwide assessment study^56^ demonstrated an undertriage rate below the ACS-COT’s 5% threshold, suggesting effective triage practices yet highlighting the necessity for ongoing scrutiny of patient injury characteristics to enhance triage precision. Conversely, Horst et al^57^ identified a significant undertriage issue in Pennsylvania, particularly for moderate to major trauma cases, underscoring the variability in triage success across regions and the potential for systemic improvements.

The concept of functional inclusivity introduced by Wohlgemut et al^58^ and the exploration of new trauma centre impacts by Ciesla et al^59^ reflect the evolving metrics for assessing trauma system performance, emphasising the importance of both geographic and resource considerations in triage decisions. The disparity in triage effectiveness between rural and urban settings, as noted by Deeb et al,^60^ alongside the mitigating role of air medical services, underscores the multidimensional challenges of providing equitable trauma care.

The outcome of prehospital triage at the injury scene could further affect subsequent patient transfers and treatment. Garwe et al^61^ built a propensity scoring model to measure the probability of direct transport, here based on the criteria of an American state-defined prehospital triage and transport guidelines, thereby finding several prehospital variables related to the high propensity of direct patient transfer to level I trauma centres, such as low Glasgow Coma Scale score, penetrating injury, traffic-related injury, closer distance between injury scene and level I trauma centre, and involvement of advanced life support emergency medical service. Despite the aforementioned factors, Sturms et al^62^ stated that direct transfer to major trauma centres is also associated with a higher ISS scale, penetrating injuries, and significant head or spine injuries. In assessing the impact of patient transfers generated by early trauma clinical prediction models on mistriage rate, Henriksson et al^63^ found that the model transfer resulted in either an increased or reduced mis-triage rate, based on the healthcare environment, and it had a greater impact on overtriage than on undertriage. Curtis et al^64^ stated that undertriaged patients spent much more time in the emergency department and suffered delays in following definitive care. Sewalt et al^65^ found that using ISS > 15 alone to define serious injury and determine the destination of the patients may lead to high overtriage rates.

Several articles also focussed on the impact of air medical care on mistriage rates, Brown et al^66^ discovered that 57% of patients who were transported by helicopter had an ISS lower than 15, suggesting that over-triage continues to affect the utilisation of helicopter emergency service. Madiraju et al^67^ further found that, although air transport occurred in only 28% of all trauma alerts, it accounted for 78% of overtriage costs during the 5 year study period in an American regional trauma system; this occurred because of complex trauma triage algorithms. Therefore, they suggested that it is necessary to revise the current trauma team activation protocols to simplify the decision-making process of air medical transportation and reduce healthcare costs. Other researchers, however, who have looked at the overtriage rate of helicopter emergency medical service (HEMS) in an Australian trauma system have found 51.1% of pre-hospital overtriage and 28.7% of secondary overtriage rate,^68^ which suggests additional research is necessary to improve HEMS dispatch criteria. Chen et al^69^ further investigated the availability of current trauma triage criteria in ACS-COT field triage guidelines for identifying patients at the injury scene who would benefit from HEMS transfer over GEMS. According to the result, HEMS transport is beneficial for patients with an aberrant prehospital RR, a prehospital GCS score of 8 or below, and haemothorax or pneumothorax.

In addition to the above assessment of pre-hospital triage on patient outcomes, several studies focussed on secondary triage evaluation in the American trauma system. Similar to prehospital triage, accurate secondary triage (with the goal of minimising both overtriage and undertriage rates) is crucial for both patient outcomes and resource use. Osen et al^70^ found that, although the system’s general secondary overtriage rate is modest, there is a considerable variation in secondary triage accuracy between non-trauma centres and the lowest-level trauma centres, which is not entirely explained by the hospital’s case mix or resources without considering other adjustable variables, such as institutional protocols and structures.^71^ Besides, Osen et al^70^ revealed that paediatric patients experience considerably more overtriage than adult patients, and insurance is likely to act as a barrier for appropriate interfacility transfer. In contrast, a more detailed study^72^ of the impact of secondary triage on subsequent treatment (eg, need for ICU admission, surgical intervention, mortality, and early discharge) has found that, although there appears to be a positive association with secondary overtriage in terms of injury pattern, originating centre, and insurance status, specific drivers are yet to be identified. Moreover, Rivara et al^73^ found a minimal increase in the risk-adjusted mortality compared with long-term survival (within 50-365 days) for patients referred to a trauma centre after receiving secondary triage and those transferred directly from the injury scene, suggesting that secondary triage may not be a significant independent predictor of survival among major and moderate trauma patients. Whereas a similar comparison for in-hospital mortality conducted by Nirula et al^74^ found the secondary triage of major trauma patients is related to an increased risk of mortality, which is probably driven by a delay in the management and control of definitive care. A more recent study^75^ identified the survival benefits for particular patients (pelvic fracture, penetrating mechanism, etc.) linked with the secondary transfer. Further underscoring the systemic implications of triage, a study by Shi et al^76^ found that state trauma funding is positively associated with higher rates of re-triage and decreased in-hospital mortality for severely injured patients. The research indicated that retriage acts as a crucial moderator, suggesting that states with dedicated trauma funding can support more efficient and effective re-triage systems.

#### Identification of triage predictors

Research has made progress in identifying the characteristics that predict triage outcomes. Madiraju et al,^67^ Curtis et al^64^ and Jensen et al^42^ identified various predictors of overtriage and undertriage, such as patient demographics, injury severity, and clinical indicators. These findings not only improve our understanding of triage dynamics but also open the way for developing more sophisticated triage protocols capable of adapting to the complexities of patient presentations.

Expanding the range of predictors within trauma triage, recent research by Cohen et al^77^ identifies critical clinical markers – specifically, advanced age, certain physiological measurements like GCS score and systolic blood pressure, and respiratory distress – as significant indicators for acute care necessity following major trauma. These insights contribute to an expansive collection of data-driven predictors, emphasising the necessity of developing advanced triage protocols that integrate a complex combination of patient characteristics, clinical metrics, and injury severity to enable more precise resource allocation in trauma systems.

Advances in computational models and data analytics, as evidenced by the studies,^78,79^ demonstrate the potential of integrating machine learning and artificial intelligence such as Random Forecast and eXtreme Gradient Boosting into the triage process. By employing complex algorithms and comprehensive datasets, these models offer a promising avenue for refining triage decisions, aiming to reduce both overtriage and undertriage rates. This approach signifies a shift towards more data-driven, personalised trauma care, aligning with the broader goal of enhancing the responsiveness and efficacy of trauma systems in meeting the urgent needs of injured patients.

### Timeliness of in-hospital intervention

The journey of a patient through a trauma network encompasses a series of in-hospital interventions, including patient transfer, diagnostic procedures, and admission. The cumulative duration of these events, from transferring to a specific healthcare facility to eventual discharge or readmission, is incorporated in the Length of Stay (LOS). Therefore, LOS emerges as the ultimate reflection of the timeliness and efficiency of in-hospital interventions within the trauma care continuum. All studies investigating patient volume and length of stay are summarised in Table 6 in the Supplemental Materials.

Moore et al^80^ emphasised the significance of monitoring the overall LOS within trauma facilities, including ICU and intermediate care wards, as a comprehensive indicator of the treatment process. ICU durations, in particular, are highlighted for their potential to extend resource use beyond expectations, thereby increasing system strain. Subsequent analyses, such as those by Moore et al^81^ and Kuimi et al,^82^ elucidate the variances in LOS across different trauma systems, attributing these differences to clinical interventions, patient complications, and discharge destinations, further complicating the landscape of trauma care delivery.

The Study by Morgan et al^83^ identified key predictors of extended LOS in trauma patients: severe injuries, reintubation, major surgeries, and specific injuries like abdominal gunshot wounds. These factors, which are indicative of high resource use, underscore the necessity for targeted management strategies that could reduce the LOS and improve the efficiency of trauma care delivery.

Within this context, a number of trauma quality improvement programmes have assessed the timeliness of clinical interventions within hospital settings across trauma networks. The studies by Haslam et al^84^ and Havermans et al^85^ show that trauma care times decreased after the infrastructure in the trauma network was improved. This included times for transfers and important procedures like CT scans and surgery. Moreover, Metcalfe et al^86^ and Moran et al^87^ indicated a similar landscape of trauma care improvements, documenting significant advancements in consultant-led treatment and a reduction in secondary patient transfers. These cumulative efforts in trauma care infrastructure and process optimisation have been shown to expedite treatment and diagnostic timelines, contributing to LOS reductions across trauma networks. These studies showed that streamlined operational processes and advanced clinical interventions are very important for reducing the LOS.

Building on the LOS foundation as a key performance indicator, the broader dynamics of patient flow within the trauma system also warrant close examination. The introduction of trauma systems has led to increased patient admission volumes,^88,89^ highlighting the critical role of such systems in enhancing accessibility to trauma care. Additionally, Durston et al^90^ showed patterns in trauma admissions related to calendar months, days, and local events at a level I trauma centre, emphasising the role of external factors in fluctuating patient volumes. This insight is crucial for resource allocation and preparing for high-demand periods, directly impacting the timeliness of in-hospital interventions and potentially reducing the LOS. External factors, including time of day, demographic trends, and weather conditions, significantly influence trauma incident frequency and admission rates, underscoring the importance of adaptive planning in managing fluctuations in demand.^91-93^ The complexity of managing patient flow is further exemplified in the strategies employed for inter-facility transfers. The decision to transfer patients is influenced by resource availability and patient-specific needs, reflecting a sophisticated coordination effort within trauma networks.^94-96^ In particular, Lin et al^97^ used discrete choice modelling to look into how important clinical and demographic factors are when deciding to transfer major trauma patients between hospitals. This shows that emergency physicians need to put more weight on some clinical signs than others when making transfer decisions.

In this continuum of care, readmission rates gain prominence as an essential metric for evaluating trauma care effectiveness. Studies have shown that unplanned readmissions are important indicators of post-discharge outcomes and system performance.^98-100^ These studies revealed that factors such as discharge destination, patient age, injury mechanism, and the initial LOS significantly contribute to the likelihood of readmission, offering crucial insights for improving patient management strategies and reducing preventable readmissions.

Moreover, innovations in predictive analytics, particularly through AI, present a frontier in optimising patient flow and resource management within trauma systems. Stonko et al^101^ and Dennis et al^102^ have pioneered models that leverage weather conditions and patient admission data to forecast trauma volumes and acuity levels. These models not only show that it is possible to predict trauma admissions, but they also suggest a way to plan for times when demand will be high. This way, trauma centres can plan ahead and make the best use of their resources and workflows. Furthermore, Stonko et al^103^ developed a machine-learning model forecasting prolonged LOS in trauma patients by utilising the data accessible upon admission. This model demonstrates the accuracy in identifying patients who are at risk of prolonged stays in the hospital, suggesting that early detection could facilitate improved resource management and discharge planning.

The effective management of the LOS, along with the strategic handling of patient flow, is crucial for establishing a responsive and efficient trauma care system. These collectively guarantee that trauma systems can effectively address the immediate requirements of patients, such as secondary transfer or readmission, while upholding exceptional standards of care quality and operational efficiency.

## Outcome

According to a systematic review of trauma systems over the past 20 years, Celso et al^104^ suggested that techniques for measuring the operational performance of trauma systems should evolve in parallel with their maturity. Published research evaluated the trauma system, mainly focussing on mortality rate and disregarding demographic data or injury severity, resulting in biased measurement of the care process for severely injured patients. They believe that process issues (eg, complexity rates, delays in treatment, or management of comorbidity), functional returns, and cost savings could be utilised as more precise performance indicators for evaluating trauma systems. The studies reviewed in this paper found that patient outcomes in the trauma network could be summarised into 3 categories: medical outcomes, functional outcomes, and patient satisfaction. Medical outcomes are associated with operations done, medical treatment regimens, and treatment-related or implanted complications. Functional outcomes assess the patient’s level of physical functioning and return to daily activities. Satisfaction measures the degree to which expectations are satisfied.^105^

### Medical outcome

#### Observation of the mortality rates

Mortality is one of the most intuitive indicators for assessing the performance of trauma networks, as evidenced by research conducted across many countries that observed its improvement. In Australia, a study by Dinh et al^106^ reported a notable reduction in both crude and risk-adjusted mortality following a quality improvement programme, underscoring the potential for systemic interventions to enhance patient outcomes. However, research by Gomez et al^107^ and Cameron et al^108^ pointed to significant variability in mortality rates across regions, indicating that case mix alone cannot fully explain these differences, suggesting a need for nationwide consistency in trauma care quality. In Canada, a significant reduction in the overall unadjusted mortality rate – 1.9% over 9 years – was observed within a provincial trauma system following the implementation of an all-inclusive system, highlighting the system’s effectiveness.^109^ However, Hameed et al^110^ and Moore et al^111^ identified a substantial variance in mortality rates across Canadian trauma centres, with disparities largely attributed to uneven access to the trauma services in rural areas. Additionally, Moore et al^112^ demonstrated that utilising a hierarchical logistic regression model for assessing risk-adjusted mortality provides more stable and reliable estimates compared with conventional logistic regression models. This approach enhances the accuracy of mortality assessments across various trauma centres, offering a more nuanced understanding of system performance and areas for improvement. In the U.S., Ashley et al^113^ and He et al^114^ found that designated trauma centres and effective rationalisation significantly impact survival rates, hence showing the need for a collaborative approach instead of merely increasing the number of high-level trauma centres. Similarly, Vernon et al^115^ and Holena et al^116^ emphasise the importance of trauma service coverage and the timing of mortality assessment in improving patient outcomes. In a Scottish population-based investigation of trauma mortality trends, the standardised mortality ratio of major trauma patients has remained stable despite rising trauma admissions.^117^ Waalwijk et al^118^ demonstrate that transferring severely injured patients from lower to higher-level trauma centres significantly lowers 24-hour and 30-day mortality rates, especially for those with critical injuries.

Recent studies further highlighted the effectiveness of organised trauma systems. In South Korea, a national trauma system was shown to significantly reduce preventable trauma death rates,^119^ and in Denmark, slight improvements in 30-day mortality rates were observed, even for severely injured patients (ISS > 15), here when adjusted for demographic factors.^120^ These outcomes suggest that systematic improvements and direct admissions to trauma centres are crucial for enhancing survival rates. Boyd et al^121^ explored the efficacy of quality indicators (QIs) on patient outcomes within trauma centres. They found no significant correlations between the use of QIs – encompassing report cards and internal and external benchmarking – and either mortality rates or complication incidences, challenging the presumed benefits of QIs in trauma care effectiveness.

Interestingly, research from Ireland^122^ and Norway^123,124^ indicated that factors such as population density and prehospital times in remote areas do not significantly impact trauma mortality rates, shifting the focus towards the quality of care and the availability of specialised services, such as orthopaedic surgery, as the key determinants of patient outcomes.

van den Driessche et al^125^ revealed no significant differences in in-hospital mortality between trauma patients primarily admitted to level I centres and those transferred from level II centres in the Netherlands, challenging the assumption that higher-level centres always yield better outcomes. This highlights that factors like age and neurotrauma severity crucially influence the need for transfers and survival, suggesting a more critical evaluation of trauma system effectiveness beyond merely expanding high-level trauma centres.

The role of the trauma centre level in patient survival is nuanced. Van Ditshuizen et al^126^ demonstrated significant benefits for severely injured patients treated at Level I trauma centres, especially for those with traumatic brain injuries or haemodynamic instability, underscoring the importance of specialised care in high-level centres for improving outcomes. Contrasting the expansion of high-level trauma centres (HLTCs), Amato et al^127^ found that a 31% increase in HLTCs over 15 years only marginally improved access and coincided with a rise in age-adjusted injury mortality rates, challenging the assumption that increasing the number of HLTCs directly correlates with enhanced access to care or reduced mortality. This points to the necessity for strategic placement and resource allocation within trauma care systems to truly benefit population health.

In addition to intuitive observational analyses of patient mortality, various studies have explored the reasons behind the variability in mortality rates among different trauma centres within trauma systems. Investigations reveal that a multitude of factors contribute to this variability, including access to trauma services, the effectiveness of specialised trauma services, and the methodologies used for risk-adjusted mortality assessment.

For instance, Wong et al^128^ and Davenport et al^129^ demonstrated that the implementation of specialised trauma services led to significant reductions in mortality rates in Austria and the UK, respectively. This suggests that the quality of care, particularly when enhanced by specialised services, plays a crucial role in improving patient outcomes. Similarly, audits of the German trauma network highlighted the positive impact of integrating different levels of trauma centres and establishing consistent standards of trauma treatment on the quality of care.^130,131^ Furthermore, Haas et al^132^ discovered that trauma centres with lower mortality rates had 30% fewer complications than those with higher mortality, emphasising the need for early complication identification and prevention. This finding aligns with suggestions by Heaney et al^133^ and Ang et al^134^ to refine risk-adjusted mortality calculations and employ patient safety indicators from the Agency for Healthcare Research and Quality (AHRQ) for benchmarking purposes.

Adding to this discourse, Plurad et al^135^ challenged previous assumptions about the disparity in patient outcomes between Level I and Level II trauma centres. Their findings indicate no significant mortality difference between severely injured patients treated at either level of trauma centre.

Furthermore, the incorporation of methodologies for risk-adjusted mortality assessment and the use of comprehensive outcome measures, such as 6-month mortality rates,^136^ alongside the identification of preventable errors in care delivery,^137^ highlight the nuanced challenges of trauma care delivery. These challenges include not only the need for high-quality in-hospital care but also the importance of efficient prehospital interventions to prevent early mortality.

Collectively, these studies demonstrate the wide range of factors that influence the differences in mortality rates across trauma centres. From the structural aspects of care delivery to the effectiveness of specialised services and the evolution of care standards, each element plays a significant role in shaping patient outcomes. The insights from Plurad et al^135^ further emphasise the evolving nature of trauma care standards, reinforcing the need for continuous improvement and strategic planning within trauma care systems to enhance patient survival rates across all levels of care.

#### Predictors for mortality forecasting

Evaluating trauma system performance typically involves comparing the expected and observed mortality rates, leading to significant research into identifying the predictors of mortality and developing forecasting methods.

##### Transportation mode

Among the variables, the mode of transport for trauma patients is a frequently examined predictor. A 10-year longitudinal study by Andruszkow et al^138^ suggested that HEMS are a positive independent predictor of survival for major trauma patients. This finding is supported by Brown et al,^139^ who emphasised the prognostic value of helicopter transport for patients with an ISS above 15. However, the benefits of air medical services in improving patient survival have been debated. Literature focussing on HEMS often segregates patients into ground versus air transport and evaluates mortality differences while accounting for various confounders.

Univariate analyses, which consider only the mode of transport’s influence on mortality, have presented mixed results. For instance, Ryb et al^140^ observed a higher mortality rate for HEMS patients than ground transport. Similarly, Beaumont et al^141^ found no significant survival benefit from HEMS over ground emergency medical services (GEMS). In contrast, Sborovet al.^142^ reported that the introduction of HEMS in rural areas significantly reduced mortality rates.

Multivariate analyses, which consider various confounders, reveal the conditional effects of HEMS on mortality. Sullivent et al^143^ discovered that the mortality reduction from HEMS was significant only in trauma patients aged 18 to 54, not in older patients. The study by Rhinehart et al^144^ indicated that the benefit of HEMS was limited to patients within a specific distance from trauma centres. Tsuchiya et al^145^ observed that patients with an ISS over 16 or those suffering from specific types of injuries had significantly lower mortality rates with helicopter transport. The authors estimated that HEMS saved between 2.3 and 6.5 lives per 100 dispatches for major trauma patients.

Despite these findings, some studies have found no significant survival benefit from HEMS when accounting for potential confounders such as helicopter availability, transfer times, patient characteristics, and injury mechanisms. For example, Shaw et al^146^ and Cudnik et al^147^ did not observe improved survival with HEMS, aligning with de Jongh et al,^148^ who reported that HEMS was weakly associated with increased in-hospital mortality for patients with traumatic brain injury (TBI) and decreased mortality for non-TBI patients. This highlights the complexity of the impact of HEMS, suggesting that, although it can enhance early survival for TBI patients, it may not translate into reduced in-hospital mortality. Adding to this complex narrative, Karrison et al^149^ examined an urban trauma network and found that, after adjusting solely for injury severity, the driving distance and EMS transfer time had a modest but linear impact on mortality across a range of 0 to 12 miles. This suggests that logistical considerations, such as transport time and distance, play a role in patient outcomes independent of transport mode.

Overall, the relationship between transport mode and trauma outcomes is complex. Although HEMS can offer survival advantages under certain conditions, its effectiveness is influenced by numerous factors, including patient age, proximity to trauma centres, and specific injury characteristics. These findings highlight the necessity for individualised transport decisions based on a comprehensive assessment of each trauma case.

##### Process indicators

In trauma systems, the determinants of mortality extend beyond transport methods to encompass various process indicators, such as patient arrival patterns, the LOS, and the timeliness of care. To provide additional insight, studies that applied these process indicators as predictors to interpret or forecast mortality are summarised in Table 7.

Gallagher et al^150^ examined the influence of pre-transfer CT scans at lower-level facilities on patient outcomes at level I trauma centres, revealing a surprising survival advantage despite prolonged transport times. Similarly, Little et al^151^ found that admission to a trauma network during weekends, particularly Fridays and Saturdays, correlates with an increased 30-day mortality rate for patients with moderate to severe injuries.

Adding to this complexity, Tiruneh et al^152^ discovered that severely injured patients undergoing inter-hospital transfers face higher mortality rates, underscoring the critical need for optimising prehospital triage and the transfer process, especially for the elderly and critically injured. Similarly, Waalwijk et al^118^ demonstrated the life-saving potential of secondary transfers from lower-level to higher-level trauma centres, which significantly lower both 24-hour and 30-day mortality rates. Their findings emphasise the importance of an efficient transfer protocol in the trauma care continuum, enhancing survival rates for critically injured patients.

Contrasting views, however, call into question the significance of these process indicators. Hill et al^153^ presented a systematic review showing negligible differences in mortality between patients transferred from other facilities versus those directly admitted to major trauma centres. This study raised concerns about the potential underestimation of the impact of transfer status due to the exclusion of patients who did not survive the transfer. In another vein, Calland and Stukenborg^154^ found the patient volume to be a non-predictive factor for in-hospital mortality across various trauma centres. This was reinforced by Sewalt et al,^155^ who reported no association between hospital volume and in-hospital mortality or the LOS, except for a longer intensive care unit ICU LOS without referrals. This finding diverges from the research by Minei et al,^156^ which suggested that higher trauma centre volume could lead to decreased mortality.

Further research by Byrne et al^157^ posited EMS prehospital time as being an independent predictor of ED mortality yet found no correlation with risk-adjusted mortality in trauma centres. Berkeveld et al^158^ also observed no significant association between prehospital time and mortality within a Dutch trauma system, likely because of the short distances involved and the swift transfer to level I trauma centres.

These studies have presented a complex landscape where some process indicators might have a nuanced role in mortality outcomes, and others may not significantly affect survival. Although some evidence has suggested benefits associated with certain prehospital and hospital processes, other research has challenged the universality of these findings, advocating for a more discerning evaluation of their impact on mortality. The mixed results demonstrate the intricacies of healthcare delivery within trauma networks, underlining the necessity for a multifaceted approach when predicting outcomes.

##### Trauma scoring systems

The development and refinement of composite scoring systems form a substantial part of mortality prediction research in trauma care, integrating both prehospital and in-hospital variables. Javali et al^159^ catalogued existing trauma scoring systems, analysing the predictive accuracy of 4 widely used models (Table 8 in the Supplemental Materials).

Their findings highlight that, although the Revised Trauma Score (RTS) utilises prehospital data points like the Glasgow Coma Scale, respiratory rate, and systolic arterial pressure, it is only when combined with the Injury Severity Score (ISS) and age in the Trauma Injury Severity Score (TRISS) that a more precise mortality prediction is achieved.

The advancements by Napoli et al^160^ with the introduction of the Relative Mortality Metric (RMM) and Relative Mortality Performance Trend (RMPT) offer a refined approach to evaluating trauma centre performance. These metrics address the shortcomings of traditional scoring systems, such as the W-Score, especially in low acuity cases or when dealing with small sample sizes. By stratifying patients by acuity levels and tracking temporal changes, the RMM and RMPT provide a more accurate and dynamic assessment of care effectiveness across the patient spectrum.

Although the ISS has its challenges, notably a time-intensive recording process, Osler et al^161^ developed an alternative model using readily available ICD-9 codes, offering not only better discrimination but also a more streamlined calculation than the ISS. This was further supported by Fugazzola et al^162^ within an Italian regional trauma system, prompting Osler et al^161^ to advocate for the adoption of new ICD-10 codes in mortality prediction models.

The RTS, which is precise but complex for use in high-pressure prehospital settings, led to the creation of the MGAP score by Sartorius et al,^163^ which incorporates variables like the mechanism of injury, GCS, age, and arterial pressure, which outperforming RTS in predicting mortality. Rahmani et al^164^ validated the MGAP and its revision, the GAP score (which omits the mechanism of injury), both of which showed reliable performance in mortality forecasting within an Iranian trauma system.

Advancements in predictive modelling were also made by Schluter,^165^ who enhanced the TRISS model by incorporating interactions between injury mechanisms and other key variables. Gunning and Leenen^166^ further tailored the TRISS model for regional mixed trauma populations, suggesting modifications for level I trauma population predictions. Building on the correlation between base deficit and mortality,^167^ introduced the Base Deficit Injury Severity Score (BISS), which combines base deficit with ISS, yielding a model comparable in predictive capability to both TRISS and the ASCOT.

Finally, broader predictive measures have been explored, such as the Intermountain Risk Score (IMRS),^168^ which includes components of the complete blood count and basic metabolic profile along with patient demographics, hence proving to be a strong predictor of short- and long-term mortality. Miller et al^169^ modified the Rapid Emergency Medicine Score (REMS) by adjusting the weightings of certain variables, resulting in a score that was more predictive of in-hospital mortality than other scores like RTS, ISS, MGAP and Shock Index.

These studies underscored a dynamic field where trauma scoring systems are constantly evolving, with newer models seeking to streamline the predictive process while maintaining or improving accuracy. The quest for an optimal scoring system continues, reflecting the nuanced nature of trauma care and the multifactorial aspects of mortality prediction.

##### Clinical predictors

Beyond established trauma scoring systems, research has identified several clinical predictors that could potentially influence mortality outcomes. Prehospital lactate (pLA) levels, as Guyette et al^170^ notes, are an independent predictor not only for in-hospital mortality but also for urgent surgical interventions and the onset of multiple organ dysfunction syndromes. Building on the significance of physiological indicators, Thompson et al^171^ emphasised that, although a multitude of field measurements could complicate prehospital triage, specific indicators like the Glasgow Coma Scale (GCS), respiratory rate (RR) and patient age are crucial for determining the necessity of transfer to major trauma centres and are closely linked to patient survival until discharge. A study from an Italian trauma centre showed trauma mortality now primarily occurs within the first hour due to care advancements, with early deaths declining and patient age, emergency care and injury severity being key mortality predictors, emphasising the importance of specialised trauma teams in reducing early fatalities.^172^

The volume of trauma centres has also been scrutinised regarding its impact on patient outcomes. Brown et al^173^ reported that shifts in trauma centre volume have a predictable effect on standardised mortality rates (SMR), with each percentage change in volume correlating with a respective increase or decrease in SMR over time. This suggests that institutional workload can be a determinant of patient survival, highlighting the importance of resource allocation in trauma care. The efficacy of mortality prediction tools has been further explored, with Dinh et al^174^ investigating the relationship between the estimated ISS from the Abbreviated Injury Scale to International Classification of Disease (AIS-ICD) and the TRISS. Their findings indicated a substantial correlation between the volume of major trauma, as gauged by the AIS-ICD mapping, and the predictive accuracy of TRISS for mortality.

Finally, Balvers et al^175^ identified hypothermia – defined as a body temperature below 35°C – as a significant and prevalent factor among major trauma patients, showing that it is closely associated with mortality both within the first 24 hours and at 28 days post-injury. This underscores the critical nature of temperature regulation as part of the initial management in trauma cases.

Together, these studies broaden the scope of mortality predictors to include physiological metrics and institutional factors, offering a composite picture of the myriad of influences that can dictate patient outcomes in trauma situations. These findings support the continuous refinement of triage and treatment protocols to incorporate a wide array of clinical data and environmental factors, ensuring a more comprehensive and effective response to trauma care.

### Functional outcome and patient satisfaction

Functional outcomes and patient satisfaction are pivotal in assessing the effectiveness of trauma care systems. These measures reflect not only immediate recovery but also long-term quality of life (QoL) and integration back into society. Nirula and Brasel^176^ demonstrated that higher-tier trauma centres significantly enhance functional outcomes, particularly in patients with minimal penetrating injuries, underscoring that the advanced care capabilities of these centres notably contribute to patient independence.

Ardolino et al^177^ categorised outcome measures into 4 domains: basic functional outcomes, quality of life (QoL), return to work and education, and patient experience. They advocated for the broad applicability of functional and QoL indicators but note the lack of quantification for work return and patient experience measures. Despite this, the early establishment of a UK trauma network did not show a mortality benefit within 6 months; however, Metcalfe et al^178^ observed a higher rate of patients discharged with favourable recovery codes, indicating early functional outcome benefits.

Long-term disability predictors, such as ISS, age, and initial GCS scores, were identified by Martino et al.^179^ Complementary analysis by Gunning et al^180^ expands on this, suggesting patient and injury characteristics as predictive of health-related quality of life (HRQoL), thereby offering a comparative basis for observed outcomes.

Hung et al^181^ demonstrated the long-term health impacts of trauma, noting significant challenges in both functional recovery and overall health status for up to 7 years post-injury. They stress the necessity for continuous monitoring and support due to sustained declines in the physical and mental well-being of trauma survivors. Similarly, van Ditshuizen et al^182^ explored health-related quality of life (HRQoL) and return to work 1 year post-major trauma, emphasising persistent cognitive challenges and a 68% return-to-work rate. Their findings highlight the need for comprehensive recovery strategies that integrate both physical and psychological support within trauma networks. Building on these observations and analysis of long-term recovery, Jones et al^183^ investigated how service provision and geographical location affect rehabilitation outcomes for multiple trauma patients within trauma network settings. Their study reveals that despite the benefits of regional trauma networks in reducing mortality, significant recovery challenges persist, particularly in meeting rehabilitation needs and ensuring service accessibility. It highlights the critical need for enhanced communication and coordination within trauma networks to facilitate better recovery outcomes. This study not only focuses on the evaluation of rehabilitation services’ effectiveness based on location but also emphasises the necessity for structured rehabilitation support in the trauma care continuum, contributing to the literature on patient experiences and outcomes post-trauma.

Collectively, these studies highlighted the complexity of trauma recovery and the importance of early indicators for predicting long-term patient outcomes. They underscore the necessity of sustained, multidimensional support and effective trauma network organisation to enhance functional outcomes for trauma patients. The integration of comprehensive rehabilitation and coordinated care is crucial for improving patient recovery and quality of life.

## Discussion

This study outlines the evaluation metrics employed to gauge the operational performance within trauma networks, along with an examination of the pertinent operational management methodologies. Furthermore, it underscores gaps within the recent literature concerning the assessment of certain operational metrics and delineates future research perspectives.

### Main findings and research gaps

The assessment of operational performance within trauma networks encompasses multiple phases, extending from the setting of the trauma network to its post-operation. In this context, a set of evaluation indicators has been identified, characterised by their intricate complexity.

In the structural evaluation of trauma networks, our review has identified critical areas of focus that are foundational to the operational efficacy and optimisation of trauma care systems. The optimisation of healthcare facility locations emerges as a concern, with innovative models enhancing the strategic placement of trauma centres and emergency service resources to improve response times and coverage significantly. Simultaneously, there has been a strong focus on trauma resource management due to its crucial role in facilitating efficient data management, decision-making, and operational focus within trauma units. This has a direct influence on clinical results and financial efficiency. Furthermore, the operation of Emergency Management Services (EMS) commands attention for its comprehensive challenges and solutions spanning emergency care pathways. This includes the deployment of advanced simulation models and queueing theories to optimise staff allocations and patient flow, directly influencing the resilience of emergency departments to overcrowding and enhancing overall system responsiveness. Collectively, these structural evaluation metrics underscore the complexity of trauma network operations and highlight the necessity of integrated, multi-faceted approaches to improve trauma care delivery and patient outcomes.

The accuracy of triage stands as a crucial process evaluative metric within the pre-hospital stage of the trauma network, critically dictating the timeliness of subsequent treatment and influencing patient in-hospital mortality rates. Undertriage has been observed to exacerbate mortality by delaying the dispatch of patients with severe injuries to high-level trauma centres for comprehensive care. Conversely, overtriage may lead to an inefficient allocation of trauma system resources and reduced system inclusivity by channelling patients with minor to moderate injuries to facilities that provide the highest level of care. Identifying optimal thresholds for these rates is imperative for the efficacy of a given trauma system. Additionally, the applicability of benchmarking criteria, such as the undertriage rate of 5% and the overtriage range of 30% to 50% as recommended by the American College of Surgeons Committee on Trauma (ACS-COT), requires further investigation across diverse trauma systems. Triage performance is subject to significant variability, influenced by a multiplicity of factors, including geographic environment, demographic composition, the operational definition of injury severity, the development and implementation of triage protocols, as well as the subjective judgement exercised by emergency medical services (EMS) providers. From an operational management standpoint, the triage rate is a quantifiable criterion that can be integrated into models that simulate patient flow, such as queueing theory or system dynamics models. These models allow for a holistic view of the trauma network, illustrating how triage decisions impact patient pathways and resource utilisation throughout the system. The development of triage protocols, therefore, not only requires a clinical lens – focussing on accuracy and patient outcomes – but also an operational perspective that considers the flow and distribution of patients. It is anticipated that the evolution of triage protocols will embrace both clinically relevant variables and operationally quantifiable measures, aiming to streamline pre-hospital processes and enhance system-wide efficiency.

The patient length of stay (LOS) emerges as another pivotal process metric for appraising the operational efficacy of trauma network systems. Following triage, patients undergo a set of clinical interventions encompassing stabilisation, surgical procedures and ward admissions – which cumulatively contribute to the total LOS within the trauma care continuum. Nevertheless, a review of extant literature reveals a lack of comprehensive evaluation of LOS across trauma networks. Notably, analyses predominantly revolve around discrete components, such as the duration of CT scans or ICU admissions for specific trauma injuries, with scant attention to overall LOS metrics post-establishment of trauma networks. Moreover, few studies discuss the association between overall LOS and its determinant factors. From an operational systems perspective, a prolonged LOS not only exacerbates in-hospital patient volume and bed occupancy but also potentially affects the quality of patient rehabilitation outcomes post-discharge. It is imperative to recognise that LOS, while often categorised as an outcome measure, can be reinterpreted as a process indicator or an intermediate transitional outcome within a healthcare systemic framework. Within the trauma network, LOS works as a mirror to the efficacy of in-hospital care, whereas at the point of discharge, it influences subsequent rehabilitation measures and patient satisfaction levels. Future research should be orientated towards identifying the predictors that significantly impact overall patient LOS within trauma networks. Developing sophisticated forecasting models for LOS can aid in enhancing decision-making processes relevant to the allocation of trauma healthcare resources and the strategic deployment of healthcare personnel.

In the review of outcome evaluation metrics within trauma care, mortality unequivocally emerges as the predominant focus of discussion. Hierarchically organised trauma facilities have demonstrated a marked improvement in mortality outcomes, substantiating the effectiveness of the trauma system structure. Scrutiny within regional scopes has unearthed variances in performance among trauma centres, prompting adjustments in anticipated mortality rates. Beyond the in-hospital mortality rates, there is a recognition of the importance of monitoring long-term mortality benchmarks, encompassing intervals of 30 days, 6 months, and up to a year post-trauma. Moreover, the refinement of mortality forecasting models within trauma networks is in progress, with numerous trauma scoring systems – relying on anatomical and physiological predictors – undergoing continual development to augment the precision of mortality prognostications. Diverse factors have been identified as correlating with mortality outcomes, such as patient demographics, mode of transportation, transfer status, and composite clinical scores. Notwithstanding, the debate persists regarding the efficacy of helicopter rescue services over ground emergency services in reducing mortality, with studies indicating variable patient outcomes when accounting for confounders, including transport time, distance, patient characteristics, and the nature of the injury.

In contrast, the exploration of other functional outcome metrics, such as quality of life and patient satisfaction, remains conspicuously underrepresented in existing literature. Importantly, limited research currently focuses on the quality and operations management of post-trauma and post-acute phase rehabilitation. Most trauma patients suffer emotional, physical, and cognitive issues after discharge,^179^ and these irreversible impairments may inhibit their ability to return to society and daily activities, significantly affecting their quality of life. Specifically, Kingston^184^ have stated that the stiffness and loss of range of motion from traumatic hand injuries significantly impact patients’ quality of life, particularly for those in rural regions. They proposed that the implementation of a collaborative and adaptable rehabilitation programme, irrespective of residential location, is a crucial component of the therapist’s intervention plan. Moreover, Sveen et al^185^ argue that a clinical pathway providing specialised rehabilitation without delay in dedicated units could greatly enhance independence in patients with severe traumatic brain injuries. Further insights into addressing unmet rehabilitation needs are provided by Kettlewell et al,^186^ who highlight significant service gaps in vocational and psychological support following major trauma across several UK health districts. Their study underscores the inconsistency in service provision, particularly for musculoskeletal injuries, and the long wait times – up to 12 months – for community rehabilitation. This delineation of usual care and unmet needs emphasises the complexity of the trauma rehabilitation pathway and the variability in service delivery across regions, which complicates the implementation of standardised rehabilitation protocols. Overall, this research gap highlights a vital need for enhanced data collection and statistical analysis in these domains. Continuous tracking and analysing of rehabilitation information after patients’ discharge could support medical professionals from rehabilitation facilities to recognise unmet rehabilitation needs, formulate strategies and protocols for functional recovery, and consequently enhance the quality of life for trauma patients. Future research should focus on an expanded assessment of trauma patient outcomes in the post-hospital phase, extending beyond mortality to include functional recovery and overall well-being.

The literature review reveals that while analytical OM toolkits have been used to estimate admission volume, mortality rate, and length of stay (LOS), their application remains limited. Current research, in particular, frequently misses the need to quantify uncertainty when modelling demand, admissions, or LOS, which is critical for making educated decisions. Therefore, future research should consider uncertainty quantification in model outputs. Furthermore, no research has integrated modelling results with real utility indicators, such as financial or clinical outcomes. This relationship is critical since practitioners want direction from measurements that extend beyond simple statistical indicators. Our study identified only a few articles that used machine learning methodologies, notably for anticipating outcomes. While we see the need for more study in this area, future studies should look at how physician knowledge and expertise may be combined with artificial intelligence and machine learning to build hybrid AI systems.^187^ Such integration would increase the trustworthiness and potential usefulness of these models.

Finally, a crucial issue that demands attention is the reproducibility of results in this area. The lack of reproducibility is a key barrier for the trauma network and research community, especially when using analytical methods like OM. To improve reproducibility, analyses and data should be shared (when feasible) via open-access platforms.

### Limitations

A main limitation of this systematic review is that we identified few studies that discuss functional outcomes and customer satisfaction. This may be due to the fact that relevant keywords such as rehabilitation’,’ recovery’ and ‘quality of life’ were not added to the keyword set used for the search. The limited number of studies makes it impracticable to synthesise current quantitative standards and statistical analyses for outcome indicators such as quality of life and functional outcomes or the evaluation of the operational performance of rehabilitation centres within trauma networks. Additionally, the clinical pathways and processes coordinating interactions between trauma and rehabilitation facilities within trauma networks need further investigation.

## Conclusion

The development of trauma systems has significantly improved the survival rates of trauma patients. Historically, the evaluation of most trauma networks has focussed primarily on triage accuracy and hospital mortality rates. This review, however, extends the scope of evaluation metrics for trauma networks and proposes an agenda for further research. By examining the determinants of patients’ LOS within the trauma network and monitoring their rehabilitation trajectory, healthcare professionals can better allocate clinical resources, mitigate the risk of readmission, and thereby reduce the prolonged LOS and effectively plan for rehabilitation. Yet, the current understanding of overall patient flow management and long-term rehabilitation outcomes remains constrained. Future works should concentrate on examining and modelling the length of stay for patients, accounting for the uncertain nature of the problem, alongside gathering and analysing data pertinent to the patient rehabilitation process. Such initiatives are crucial for improving patient flow management within trauma networks and enriching patients’ rehabilitation experiences. By placing emphasis on these domains, substantial progress can be achieved in enhancing the optimisation of trauma care delivery and the effectiveness of the quality of patient recovery outcomes.

## Supplemental Material

sj-pdf-1-rpo-10.1177_27536351241310645 – Supplemental material for A Systematic Literature Review of Trauma Systems: An Operations Management Perspective Supplemental material, sj-pdf-1-rpo-10.1177_27536351241310645 for A Systematic Literature Review of Trauma Systems: An Operations Management Perspective by Zihao Wang, Bahman Rostami-Tabar, Jane Haider, Mohamed Naim and Javvad Haider in Advances in Rehabilitation Science and Practice
