# Supplementary material for: A Systematic Literature Review of Trauma Systems: An Operations Management Perspective
Source: Adv Rehabil Sci Pract. 2025 Jan 16;14:27536351241310645. doi: 10.1177/27536351241310645 (PMC11742173; doi:10.1177/27536351241310645)
Supplement: sj-pdf-1-rpo-10.1177_27536351241310645 – Supplemental material for A Systematic Literature Review of Trauma Systems: An Operations Management Perspective [file sj-pdf-1-rpo-10.1177_27536351241310645.pdf]

Table 2. Mixed Methods Appraisal Tool

		Screening		1. Qualitative Studies					2. Quantitative randomized controlled trials					3. Quantitative nonrandomized					4. Quantitative descriptive					5. Mixed Methods Studies					
**Reference**	**Donabedian framework dimensions**	S1. Are there clear research questions?	S2. Do the collected data allow to address the research questions?	1.1. Is the qualitative approach appropriate to answer the research question?	1.2. Are the qualitative data collection methods adequate to address the research question?	1.3. Are the findings adequately derived from the data?	1.4. Is the interpretation of results sufficiently substantiated by data?	1.5. Is there coherence between qualitative data sources, collection, analysis, and interpretation?	2.1. Is randomization appropriately performed?	2.2. Are the groups comparable at baseline?	2.3. Are there complete outcome data?	2.4. Are outcome assessors blinded to the intervention provided?	2.5. Did the participants adhere to the assigned intervention?	3.1. Are the participants representative of the target population?	3.2. Are measurements appropriate regarding both the outcome and intervention (or exposure)?	3.3. Are there complete outcome data?	3.4. Are the confounders accounted for in the design and analysis?	3.5. During the study period, is the intervention administered (or exposure occurred) as intended?	4.1. Is the sampling strategy relevant to address the research question?	4.2. Is the sample representative of the target population?	4.3. Are the measurements appropriate?	4.4. Is the risk of nonresponse bias low?	4.5. Is the statistical analysis appropriate to answer the research question?	5.1. Is there an adequate rationale for using a mixed methods design to address the research question?	5.2. Are the different components of the study effectively integrated to answer the research question?	5.3. Are the outputs of the integration of qualitative and quantitative components adequately interpreted?	5.4. Are divergences and inconsistencies between quantitative and qualitative results adequately addressed?	5.5. Do the different components of the study adhere to the quality criteria of each tradition of the methods involved?	**Comments**
Crowley et al. [71]	Process	Yes	Yes											Yes	Yes	Yes	Yes	Yes											1. Retrospective observational study using multivariate logistic regression<br>2. Operationally orientated, seeking to optimise trauma system efficiency by identifying factors associated with unnecessary transfers, which could improve patient routing and reduce costs. No direct use of OR/OM tools; the analysis informs trauma system optimisation, potentially guiding resource allocation and protocol adjustments to reduce unnecessary transfers.
Martino et al. [178]	Outcome	Yes	Yes											Yes	Yes	Yes	Yes	Yes											1. Retrospective cohort study using logistic regression<br>2. Operationally oriented, evaluating long-term outcomes to inform trauma system quality improvement and follow-up care planning. No direct use of OR/OM tools; the analysis highlights critical factors affecting recovery, supporting data-driven improvements in trauma follow-up and rehabilitation pathways.
Hewitt et al. [92]	Process	Yes	Yes																Yes	Yes	Yes	Can't tell	Yes						1. Retrospective descriptive study with the geographic, temporal distribution and trauma registry data analyses<br>2. Operationally orientated, focusing on optimising prehospital care resource allocation by identifying high-demand times and locations. No direct use of OR/OM tools; the study provides essential data-driven insights for strategic planning in trauma response, enhancing resource efficiency and patient access to care.
Haslam et al. [83]	Process	Yes	Yes											Yes	Yes	Yes	Yes	Yes											1. Retrospective cohort study using logistic regression<br>2. Operationally orientated, focusing on improving trauma system performance by identifying delays and mortality risks associated with secondary transfers. No direct use of OR/OM tools; the statistical analysis supports policy improvements aimed at optimising transfer protocols and reducing mortality in trauma networks.
Beaumont et al. [140]	Outcome	Yes	Yes											Yes	Yes	Yes	Yes	Yes											1. Propensity score matching and logistic regression<br>2. Operationally orientated, focusing on evaluating transport modes and survival outcomes, highlights a systemic approach typical in operational analysis. No direct use of OR/OM tools. The study aims to improve healthcare logistics and outcomes through data-driven insights.
Fugazzola et al. [161]	Outcome	Yes	Yes											Yes	Yes	Yes	Yes	Yes											1. Retrospective study using logistic regression<br>2. Operationally orientated, aiming to improve trauma care through effective centralisation and model validation for quality improvement. No direct use of OR/OM tools; the statistical modelling provides insights for trauma system optimisation, influencing resource allocation and care standardisation across hospital levels.

Prepared using sagej.cls

Prepared using sagej.cls

Table 2 continued from previous page

Sborov et al.[141]	Outcome	Yes	Yes	Yes	Yes	Yes	Yes	Yes						1. Retrospective cohort study employing Cox regression and interrupted time series analysis.<br>2. Operationally orientated, aimed at improving trauma system performance by reducing response time for rural patients via helicopter transport. No direct use of OR/OM tools; the statistical modelling informs decision-making on helicopter base placement, enhancing access and trauma outcomes in rural systems.
Morgan et al.[82]	Process	Yes	Yes	Yes	Yes	Yes	Yes	Yes						1. Retrospective cohort study using multilevel mixed-effects logistic regression<br>2. Operationally orientated, aiming to improve resource allocation and management for high-cost trauma cases. No direct use of OR/OM tools; the statistical modelling helps inform targeted strategies for optimising care for trauma superusers, potentially reducing length of stay and associated costs.
Tillmann et al.[70]	Process	Yes	Yes	Yes	Yes	Yes	Yes	Yes						1. Population-based observational study using mixed-effect models<br>2. Operationally orientated, focusing on optimising triage accuracy to improve patient outcomes and resource utilisation in trauma systems. No direct use of OR/OM tools; the statistical modelling contributes to understanding triage practices, potentially informing system-wide improvements in trauma care.
Dinh et al.[173]	Outcome	Yes	Yes						Yes	Yes	Yes	Yes	Yes	1. Retrospective descriptive study with trauma registry and administrative data analysis<br>2. Operationally orientated, aiming to enhance trauma system efficiency by exploring the potential of administrative data to support trauma volume estimation and mortality prediction. No direct use of OR/OM tools; The data analysis contributes to resource planning and trauma registry optimisation.
Cameron et al.[107]	Outcome	Yes	Yes						Yes	Yes	Yes	Yes	Yes	1. A nationwide observational study using trauma registry data<br>2. Operationally orientated, with a focus on informing trauma system design and policy by identifying demographic trends and care outcomes. No direct use of OR/OM tools; the analysis supports evidence-based decision-making for system optimisation and resource allocation across trauma centres in Australia.
Holena et al.[115]	Outcome	Yes	Yes	Yes	Yes	Yes	Yes	Yes						1. Retrospective cohort study using probabilistic matching and risk-adjusted models<br>2. Operationally orientated, focusing on optimising mortality benchmarking processes within trauma networks to reflect accurate trauma care quality. No direct use of OR/OM tools; the statistical analysis supports fair benchmarking practices, contributing to improved accountability and system performance in trauma care.
Vernon et al.[114]	Outcome	Yes	Yes	Yes	Yes	Yes	Yes	Yes						1. Observational cohort study using multilevel mixed-effects regression<br>2. Operationally orientated, aiming to optimise trauma system performance by evaluating the impact of accreditation on patient routing and outcomes. No direct use of OR/OM tools; the statistical analysis informs trauma system enhancements to improve access and quality of care in rural settings.
Havermans et al.[84]	Process	Yes	Yes						Yes	Yes	Yes	Yes	Yes	1. Retrospective cohort study using logistic and linear regression<br>2. Operationally orientated, aiming to enhance trauma care processes and patient outcomes through infrastructure improvements. No direct use of OR/OM tools; The data-driven evaluation informs effective trauma system design, contributing to resource planning and care optimisation.
Dennis et al.[101]	Process	Yes	Yes	Yes	Yes	Yes	Yes	Yes						1. Multicenter observational study using an artificial neural networks (ANN) forecasting model<br>2. Operationally orientated, focusing on optimising trauma centre staffing and resource allocation by forecasting trauma volume patterns. Direct use of OR/OM tools through ANN modelling to inform resource planning and improve trauma care efficiency at the systems level.
Adzemovic et al.[74]	Process	Yes	Yes	Yes	Yes	Yes	Yes	Yes						1. Retrospective cohort study using propensity score-stratified Cox proportional hazards regression<br>2. Operationally orientated, seeking to improve trauma triage and patient routing by identifying which patients benefit from transfer to advanced care. No direct use of OR/OM tools; the statistical approach aids in optimising transfer decisions to enhance resource allocation and patient outcomes in trauma systems.
Moran et al.[86]	Process	Yes	Yes	Yes	Yes	Yes	Yes	Yes						1. A longitudinal cohort study using risk-adjusted survival analysis<br>2. Operationally orientated, focusing on optimising trauma care systems to improve patient survival and streamline care processes. The use of longitudinal and interrupted time series analysis to assess performance trends supports evidence-based quality improvements in national trauma care.
Moore et al.[80]	Process	Yes	Yes	Yes	Yes	Yes	Yes	Yes						1. Retrospective multicenter cohort study using multilevel linear regression<br>2. Operationally orientated, focusing on optimising trauma care efficiency by identifying factors contributing to prolonged LOS. The application of multilevel modelling supports data-driven insights for improving resource utilisation in trauma care

Table 2 continued from previous page

Polites et al.[55]	Process	Yes	Yes	Yes	Yes	Yes	Yes	Yes						1. Observational cohort study using logistic regression<br>2. Operationally orientated, aiming to improve trauma system efficiency by identifying triage shortcomings and mortality risk factors. No direct use of OR/OM tools; the statistical analysis aids in understanding and mitigating disparities in trauma triage practices, supporting trauma system quality improvement efforts.
Gomez et al.[106]	Outcome	Yes	Yes	Yes	Yes	Yes	Yes	Yes						1. Retrospective cohort study using hierarchical logistic regression<br>2. Operationally orientated, aiming to inform quality improvement and optimise trauma system performance through external benchmarking. The use of hierarchical modelling enables targeted performance assessments, supporting data-driven quality improvements in trauma care.
Voskens et al.[45]	Process	Yes	Yes	Yes	Yes	Yes	Can't tell	Yes						1. Observational study<br>2. Operationally orientated, as it aims to improve trauma triage accuracy to reduce preventable morbidity and mortality in trauma patients. No direct use of OR/OM tools; however, statistical analysis supports protocol refinement to optimise patient routing and resource allocation in trauma care system
Miller et al.[168]	Outcome	Yes	Yes	Yes	Yes	Yes	Yes	Yes						1. Retrospective study using logistic regression and ROC analysis<br>2. Operationally orientated, aiming to improve trauma triage processes by providing a reliable, easy-to-use scoring tool for mortality risk assessment. No direct use of OR/OM tools; however, mREMS supports decision-making in trauma triage and resource allocation, enhancing operational efficiency in trauma care settings.
Thompson et al.[170]	Outcome	Yes	Yes	Yes	Yes	Yes	Yes	Yes						1. Observational study using binary logistic regression<br>2. Operationally orientated, focusing on enhancing trauma triage processes by identifying predictors for rapid intervention. No direct use of OR/OM tools; however, statistical analysis helps inform pre-hospital triage guidelines and supports decision-making in trauma care response.
Moore et al.[110]	Outcome	Yes	Yes						Yes	Yes	Yes	Can't tell	Yes	1. Retrospective cohort design with multilevel logistic regression, sensitivity analysis, and multiple imputations for missing data.<br>2. Operationally orientated but does not employ specific OR/OM tools or methodologies, focusing instead on observational and statistical methods.
Boyd et al.[120]	Outcome	Yes	Yes	Yes	Yes	Can't tell	Yes	Yes						1. Retrospective cohort design, using generalised linear mixed models<br>2. Operationally orientated and discusses quality improvement in trauma systems but does not specifically employ OR/OM tools, focusing instead on statistical analyses and benchmarking methods.
Brown et al.[172]	Outcome	Yes	Yes	Yes	Yes	Can't tell	Yes	Yes						1. Retrospective cohort design with longitudinal panel modelling and multilevel logistic regression<br>2. Operationally orientated, focusing on trauma system design and performance evaluation but not specifically applying traditional OR/OM tools, instead utilising statistical models to understand system-level impacts of volume changes on outcomes.
Ciesla et al.[58]	Process	Yes	Yes	Yes	Yes	Can't tell	Yes	Yes						1. Retrospective cohort design with statistical analysis<br>2. Operationally orientated. It does not employ specific OR/OM tools, instead focusing on descriptive and statistical methods to analyse system-level outcomes.
Gunning et al.[179]	Outcome	Yes	Yes						Yes	Can't tell	Yes	No	Yes	1. Retrospective cohort design with linear regression<br>2. Not operationally orientated, focusing on descriptive statistical analysis without the direct application of OR/OM tools. The study emphasises observational outcomes rather than system performance evaluation.
Van Rein et al.[48]	Process	Yes	Yes						Yes	Yes	Yes	Can't tell	Yes	1. Descriptive cohort approach with observational data analysis<br>2. Operationally orientated; the study does not apply specific OR/OM tools, relying instead on descriptive statistics and compliance metrics to inform potential protocol improvements.
He et al.[113]	Outcome	Yes	Yes	Yes	Yes	Can't tell	Yes	Yes						1. Retrospective cohort design using multivariate analysis<br>2. Operationally orientated; the study does not employ traditional OR/OM tools, relying instead on statistical comparisons to assess system performance.
Metcalfe et al.[85]	Process	Yes	Yes	Yes	Yes	Can't tell	Yes	Yes						1. Retrospective before-and-after observational design<br>2. Operationally orientated, focusing on system performance evaluation but not employing specific OR/OM methodologies, using descriptive and statistical methods to assess system outcomes instead.
Kieffer et al.[90]	Process	Yes	Yes						Yes	Yes	Yes	Can't tell	Yes	1. Retrospective descriptive design with statistical analysis<br>2. Operationally orientated, providing insights for trauma care resource planning based on observed admission patterns, though it does not employ OR/OM methodologies.
Jansen et al.[116]	Outcome	Yes	Yes						Yes	Yes	Yes	Can't tell	Yes	1. Retrospective cohort design with regression analysis<br>2. Not operationally orientated, as it evaluates trauma outcomes from a quality assessment perspective rather than focusing on system performance or process optimisation. The study emphasises descriptive statistical analysis rather than OR/OM methodologies.
Moore et al.[20]	All	Yes	Yes	Yes	Yes	Can't tell	Yes	Yes						1. Retrospective cohort design with statistical analysis<br>2. Operationally orientated, focusing on system performance and trauma care quality, though it does not apply OR/OM methodologies directly, relying instead on descriptive and inferential statistics.

Prepared using sagej.cls

Table 2 continued from previous page

Mckee et al. [108]	Outcome	Yes	Yes	Yes	Yes	Can't tell	Yes	Yes						1. Retrospective before-and-after design with multivariable analysis<br>2. Operationally orientated, evaluating trauma system performance, focusing on quality improvement and resource allocation without directly applying OR/OM methodologies.
Ashley et al. [112]	Outcome	Yes	Yes	Yes	Yes	Can't tell	Yes	Yes						1. Retrospective observational design with instrumental variables and multivariable analysis<br>2. Operationally orientated, assessing trauma system performance to justify resource allocation for DTCs, though it relies on statistical analysis rather than OR/OM methodologies.
Kuimi et al. [87]	Process	Yes	Yes	Yes	Yes	Can't tell	Yes	Yes						1. Retrospective cohort design with regression analysis<br>2. Operationally orientated, aiming to inform system efficiency and patient outcomes, but it does not apply specific OR/OM methodologies directly.
Kuimi et al. [81]	Process	Yes	Yes	Yes	Yes	Can't tell	Yes	Yes						1. Retrospective observational design with multivariable regression.<br>2. Operationally orientated, evaluating trauma system access and its impact on outcomes relevant for trauma system planning and resource allocation. No direct use of OR/OM methodologies. Instead, it relies on statistical methods to assess trauma care
Shawhan et al. [49]	Process	Yes	Yes	Yes	Yes	Yes	Can't tell	Yes						1. Prospective cohort design with data analysis.<br>2. Operationally orientated, focusing on improving system performance and provider satisfaction within trauma care. No direct use of OR/OM tools; using descriptive and statistical methods to evaluate triage accuracy.
Ruchholtz et al. [129]	Outcome	Yes	Yes						Yes	Yes	Yes	Can't tell	Yes	1. Descriptive data analysis<br>2. Operationally orientated, focusing on quality improvement and standardisation in trauma care without employing OR/OM methodologies, relying instead on descriptive data from quality audits.
Metcalfe et al. [177]	Outcome	Yes	Yes	Yes	Yes	Can't tell	Yes	Yes						1. A before-and-after observational design with statistical analysis<br>2. Operationally orientated, focusing on system-level changes and their effects on trauma care outcomes, though it does not use specific OR/OM tools, relying instead on descriptive and inferential statistical methods.
Ciesla et al. [43]	Process	Yes	Yes	Yes	Yes	Can't tell	Yes	Yes						1. Retrospective observational design with descriptive statistics<br>2. Operationally orientated, focusing on evaluating system-wide triage efficiency in a mature trauma system. It does not employ specific OR/OM methodologies but uses administrative data analysis to inform trauma system performance and optimisation.
Scerbo et al. [77]	Process	Yes	Yes	Yes	Yes	Yes	Yes	Yes						1. Retrospective cohort design with the Random Forest algorithm.<br>2. Operationally orientated, exploring ways to optimise trauma system efficiency through data-driven decision-making. The study applies machine learning methods to improve resource allocation and triage accuracy in trauma care.
Cameron et al. [41]	Process	Yes	Yes						Yes	Yes	Yes	Can't tell	Yes	1. Descriptive approach, reviewing best practices and guidelines in trauma triage<br>2. Operationally orientated, focusing on trauma system optimisation to improve triage accuracy. However, it does not use OR/OM methodologies, relying on descriptive analysis to outline system components and guidelines.
Gunning et al. [165]	Outcome	Yes	Yes	Yes	Yes	Can't tell	Yes	Yes						1. Retrospective observational design, applying and modifying the TRISS model in mortality prediction<br>2. Operationally orientated, aiming to improve trauma care quality by ensuring the relevance of scoring tools for performance evaluation. The study relies on statistical analysis to assess model applicability but does not directly employ OR/OM methodologies.
Minei et al. [155]	Outcome	Yes	Yes	Yes	Yes	Yes	Yes	Yes						1. Secondary analysis of prospective, applying multivariable regression to control for confounders.<br>2. Operationally orientated, focusing on trauma system optimisation. It evaluates trauma centre resource allocation without employing traditional OR/OM tools, relying on statistical analysis of volume and outcomes.
Moore et al. [79]	Process	Yes	Yes						Yes	Yes	Yes	Can't tell	Yes	1. Retrospective cohort design with linear regression modelling<br>2. Operationally orientated, focusing on improving trauma system efficiency by understanding LOS patterns and their implications for resource use. The study primarily uses statistical methods rather than specific OR/OM tools to inform quality improvement in trauma care.
Moore et al. [97]	Process	Yes	Yes	Yes	Yes	Yes	Yes	Yes						1. Retrospective cohort design and a multivariable model<br>2. Operationally orientated, focusing on trauma system efficiency and quality improvement through benchmarking. The study provides a robust quality metric that supports hospital performance evaluation without applying traditional OR/OM methodologies.
Moore et al. [18]	All	Yes	Yes						Yes	Yes	Yes	Can't tell	Yes	1. Retrospective descriptive design, quantifying structural performance through a scoring model<br>2. Operationally orientated. Focusing on supporting trauma system improvements through structured quality assessment. Statistical validation methods are applied, aligning with system-level performance assessment without traditional OR/OM techniques.

Table 2 continued from previous page

Moore et al. [19]	All	Yes	Yes											Yes	Yes	Can't tell	Yes	Yes											1. Retrospective observational approach with statistical analysis and performance indicator calculation<br>2. Operationally orientated, focusing on performance measurement within an integrated trauma system. It applies performance indicators and statistical modelling relevant to OR/OM frameworks for healthcare evaluation.
Moore et al. [17]	All	Yes	Yes											Yes	Yes	Can't tell	Yes	Yes											1. Retrospective cohort design with a comparison of the indicator average, opportunity model, and latent variable model<br>2. Operationally orientated, aiming to enhance trauma care quality assessment by identifying optimal scoring methods for trauma centre performance evaluation. It employs statistical comparisons to analyse scoring methods, aligning with OR/OM interests in performance measurement.
Gomez et al. [91]	Process	Yes	Yes											Yes	Yes	Can't tell	Yes	Yes											1. Population-based, retrospective cohort with hierarchical regression<br>2. Operationally orientated, aiming at enhancing interfacility transfer efficiency for trauma care. It applies statistical modelling for performance evaluation but does not directly utilise OR/OM techniques.
Brown et al. [138]	Outcome	Yes	Yes											Yes	Yes	Yes	Yes	Yes											1. Retrospective cohort design with logistic regression<br>2. Operationally orientated, assessing resource allocation for transport modalities in trauma systems. Statistical analysis is applied to determine helicopter transport effectiveness, aligning with OR/OM interests in resource optimisation.
Newgard et al. [44]	Process	Yes	Yes																					Yes	Yes	Yes	Can't tell	Yes	1. Quantitative analysis of qualitative observations and interviews to model cognitive reasoning in trauma triage.<br>2. Operationally orientated, aimed at improving triage efficiency through insights into EMS decision-making processes. It employs a mixed-methods approach, combining statistical data analysis with ethnographic insights to inform trauma system practices.
Davenport et al. [128]	Outcome	Yes	Yes											Yes	Yes	Yes	Yes	Yes											1. Retrospective observational design.<br>2. Operationally orientated, with an emphasis on improving trauma care outcomes through system-level changes. Statistical analysis is used to assess the impact of care improvements, aligning with healthcare quality improvement objectives.
Moore et al. [111]	Outcome	Yes	Yes											Yes	Yes	Yes	Yes	Yes											1. Retrospective cohort design with comparing ordinary linear regression and hierarchical linear regression models.<br>2. Operationally orientated, with a focus on improving hospital profiling methods to enhance trauma care assessment. Statistical comparison of OLR and HLR aligns with health system performance evaluation but does not directly utilise OR/OM methodologies.
Hameed et al. [109]	Outcome	Yes	Yes																Yes	Yes	Yes	Can't tell	Yes						1. National survey and GIS mapping<br>2. Operationally orientated, aimed at improving trauma system access. It employs GIS mapping techniques to identify gaps in service but does not incorporate traditional OR/OM tools.
Brown et al. [65]	Process	Yes	Yes											Yes	Yes	Yes	Yes	Yes											1. Retrospective analysis using logistic regression<br>2. Operationally orientated, examining the effectiveness of helicopter transport in trauma systems. Statistical analysis is employed to assess helicopter transport's impact on survival, contributing to trauma resource allocation strategies.
Osen et al. [69]	Process	Yes	Yes											Yes	Yes	Yes	Yes	Yes											1. Retrospective analysis using multivariate regression<br>2. Operationally orientated, focusing on improving interhospital transfer efficiency and reducing unnecessary transfers. Statistical methods assess secondary overtriage patterns, informing potential areas for system improvements.
Nirula et al. [73]	Process	Yes	Yes											Yes	Yes	Yes	Yes	Yes											1. Secondary analysis of a multicenter cohort using multivariate logistic regression<br>2. Operationally orientated, aiming to improve trauma triage efficiency. The study applies statistical analysis to evaluate transport pathways, informing trauma system protocols.
Ocak et al. [47]	Process	Yes	Yes											Yes	Yes	Yes	Yes	Yes											1. Retrospective analysis using logistic regression<br>2. Operationally orientated, focusing on improving trauma triage accuracy. Statistical methods assess model performance, contributing to more efficient prehospital trauma identification.
Barringer et al. [94]	Process	Yes	Yes											Yes	Yes	Can't tell	Yes	Yes											1. Retrospective observational design with statistical analysis, including the TRISS score and W statistic.<br>2. Operationally orientated, focusing on system-level effects of hospital designation and resource utilisation but not applying specific OR/OM tools.
Sturms et al.	Process	Yes	Yes											Yes	Yes	Yes	Yes	Yes											1. Retrospective observational study<br>2. Operationally orientated, focused on optimising trauma triage and care within the Dutch trauma system. Statistical analysis evaluates triage performance but does not utilise specific OR/OM methods.
Esposito et al. [93]	Process	Yes	Yes											Yes	Yes	Yes	Yes	Yes											1. Retrospective observational study using logistic regression<br>2. Operationally orientated, aiming to identify nonclinical drivers of patient transfers and potential impacts on trauma centre efficiency. Statistical analysis is used to examine transfer patterns, contributing to system-level trauma management insights.

Prepared using sagej.cls

Prepared using sagej.cls

Table 2 continued from previous page

Sewalt et al. [64]	Process	Yes	Yes	Yes	Yes	Can't tell	Yes	Yes	1. Retrospective observational analysis using logistic regression<br>2. Operationally orientated, aimed at optimising triage protocols. Statistical models are applied to assess patient outcomes, supporting trauma system decision-making.
Berkeveld et al. [157]	Outcome	Yes	Yes	Yes	Yes	Can't tell	Yes	Yes	1. Retrospective cohort design with multivariable logistic regression<br>2. Operationally orientated, focusing on trauma system performance and response efficiency. It does not employ specific OR/OM tools but uses statistical methods to assess the impact of transport times on survival.
Wohlgemut et al. [57]	Process	Yes	Yes	Yes	Yes	Can't tell	Can't tell	Yes	1. Retrospective geospatial observational design<br>2. Operationally orientated, focusing on trauma network efficiency and resource utilisation, but it does not employ specific OR/OM tools, relying on geospatial and descriptive statistical analysis.
Deeb et al. [59]	Process	Yes	Yes	Yes	Yes	Yes	Yes	Yes	1. Retrospective observational study using logistic regression<br>2. Operationally orientated, with a focus on improving triage protocols in rural areas to enhance trauma care outcomes. Statistical analysis identifies risk factors and potential mitigation strategies relevant to trauma system optimisation.
Karrison et al. [148]	Outcome	Yes	Yes	Yes	Yes	Yes	Yes	Yes	1. Retrospective observational analysis using logistic regression and instrumental variable methods<br>2. Operationally orientated, focused on optimising emergency medical response. The study uses advanced statistical models to evaluate transport impact, aiding in trauma system planning.
Jammula et al. [53]	Process	Yes	Yes	Yes	Yes	Yes	Yes	Yes	1. Retrospective analysis using multilevel logistic regression<br>2. Operationally orientated, focusing on evaluating triage benchmarks for trauma centres to optimise patient outcomes. Statistical methods are applied to assess the impact of undertriage on survival.
Stonko et al. [100]	Process	Yes	Yes	Yes	Yes	Yes	Can't tell	Yes	1. Retrospective study using ANN<br>2. Operationally orientated, aiming to enhance trauma centre resource allocation and planning through AI-based predictions. Statistical validation supports the model's reliability for trauma volume forecasting.
Byrne et al. [156]	Outcome	Yes	Yes	Yes	Yes	Yes	Yes	Yes	1. Retrospective ecological study using multilevel logistic regression<br>2. Operationally orientated, with a focus on evaluating EMS times for potential inclusion in trauma centre benchmarking. Statistical models are applied to assess the impact of transport time on ED outcomes, relevant to trauma system management.
Heaney et al. [132]	Outcome	Yes	Yes	Yes	Yes	Can't tell	Yes	Yes	1. Retrospective cohort study<br>2. Operationally orientated, focusing on improving trauma benchmarking accuracy through refined patient inclusion criteria. The study applies statistical comparisons to evaluate model biases, supporting trauma system quality improvement.
Horst et al. [56]	Process	Yes	Yes	Yes	Yes	Yes	Yes	Yes	1. Retrospective observational study using geospatial mapping<br>2. Operationally orientated, focused on optimising trauma care access by identifying regions with high undertriage rates. Statistical and spatial analysis supports improvements in the strategic trauma system.
Chen et al. [68]	Process	Yes	Yes	Yes	Yes	Yes	Yes	Yes	1. Retrospective analysis using propensity score matching and logistic regression<br>2. Operationally orientated, with a focus on enhancing trauma triage protocols by identifying patients who may benefit from HEMS despite longer transport times. Statistical analysis supports evidence-based improvements in trauma care triage systems.
Ruchholtz et al. [130]	Outcome	Yes	Yes	Yes	Yes	Yes	Can't tell	Yes	1. Descriptive analysis of certification processes, quality metrics, and patient outcomes<br>2. Operationally orientated, focusing on trauma network efficiency and care standardisation without employing specific OR/OM tools, relying instead on quality management metrics and descriptive statistics.
Wong et al. [127]	Outcome	Yes	Yes	Yes	Yes	Can't tell	Yes	Yes	1. Retrospective cohort analysis using logistic regression<br>2. Operationally orientated, focusing on improving trauma system organisation and survival outcomes through specialist intervention. No direct use of OR/OM tools. Statistical models are applied to evaluate mortality trends relevant to trauma service enhancement.
Taylor et al. [67]	Process	Yes	Yes	Yes	Yes	Yes	Yes	Yes	1. Retrospective observational study<br>2. Operationally orientated, focusing on resource allocation and cost analysis for trauma services. No direct use of OR/OM tools; statistical methods are applied to assess financial implications and over-triage patterns.
Shafi et al. [135]	Outcome	Yes	Yes	Yes	Yes	Yes	Yes	Yes	1. Retrospective observational study using Kaplan-Meier survival curves and Cox proportional hazards models<br>2. Operationally orientated, aimed at improving trauma care quality by assessing long-term survival. No direct use of OR/OM tools; statistical analysis is applied to monitor patient outcomes, highlighting potential gaps in post-discharge care.
Guyette et al. [169]	Outcome	Yes	Yes	Yes	Yes	Yes	Yes	Yes	1. Retrospective observational study using logistic regression<br>2. Operationally orientated, with a focus on improving trauma triage and patient stratification based on pLA levels. No direct use of OR/OM tools; statistical models assess the utility of a biomarker in clinical decision-making.

Table 2 continued from previous page

Haas et al. [131]	Outcome	Yes	Yes	Yes	Yes	Yes	Yes	Yes	1. Retrospective observational study using logistic regression<br>2. Operationally orientated, focusing on identifying processes that improve trauma care quality. No direct use of OR/OM tools; statistical methods are used to compare performance metrics and highlight quality improvement opportunities.
Sartorius et al. [162]	Outcome	Yes	Yes	Yes	Yes	Yes	Yes	Yes	1. Prospective observational study using logistic regression<br>2. Operationally orientated, aiming to improve prehospital triage accuracy in trauma care. No direct use of OR/OM tools; statistical methods evaluate the MGAP score's efficiency in identifying high-risk patients for appropriate trauma centre referral.
Rivara et al. [72]	Process	Yes	Yes	Yes	Yes	Yes	Yes	Yes	1. Retrospective cohort analysis using Cox proportional hazards models<br>2. Operationally orientated, aiming to inform trauma centre triage and transfer protocols. No direct use of OR/OM tools; the study relies on statistical survival analysis to evaluate patient outcomes.
Magnone et al. [187]	Process	Yes	Yes	Yes	Yes	Yes	Yes	Yes	1. Retrospective observational study using logistic regression<br>2. Operationally orientated, with a focus on improving field triage protocols. No direct use of OR/OM tools; the study relies on statistical analysis to identify areas for triage protocol refinement.
Sturms et al. [61]	Process	Yes	Yes	Yes	Yes	Yes	Yes	Yes	1. Retrospective cohort study using logistic regression<br>2. Operationally orientated, aiming to improve triage protocols within the trauma system. No direct use of OR/OM tools; statistical analysis highlights areas for enhancing
Faul et al. [95]	Process	Yes	Yes	Yes	Yes	Yes	Yes	Yes	1. Retrospective analysis using generalised linear models<br>2. Operationally orientated, with a focus on optimising trauma resource allocation. No direct use of OR/OM tools; statistical models are employed to assess the impact of infrastructure and patient characteristics on trauma centre utilisation.
Vachon et al. [98]	Process	Yes	Yes	Yes	Yes	Yes	Yes	Yes	1. Retrospective analysis using logistic regression<br>2. Operationally orientated, focusing on improving patient follow-up and discharge protocols to reduce readmissions. No direct use of OR/OM tools; statistical analysis is employed to identify risk factors and preventable readmission causes.
Calland and Stuken-borg [153]	Outcome	Yes	Yes	Yes	Yes	Yes	Yes	Yes	1. Retrospective analysis using weighted hierarchical generalised linear models<br>2. Operationally orientated, assessing the efficacy of volume-based standards in trauma centre performance. No direct use of OR/OM tools; statistical models are employed to explore the impact of patient volume on mortality, offering insights for trauma system improvement.
Haider et al. [52]	Process	Yes	Yes	Yes	Yes	Yes	Yes	Yes	1. Retrospective observational study using logistic regression<br>2. Operationally oriented, aimed at improving trauma triage protocols by evaluating a modification to the NTTP. No direct use of OR/OM tools; statistical methods assess the impact of shock index on triage performance, potentially informing triage protocol updates.
Nirula and Brasel [175]	Process	Yes	Yes	Yes	Yes	Yes	Yes	Yes	1. Retrospective analysis using multivariate logistic regression<br>2. Operationally orientated, focused on evaluating trauma system performance in functional outcomes across trauma centre levels. No direct use of OR/OM tools; statistical models assess the impact of trauma centre levels on functional independence.
Garwe et al. [60]	Process	Yes	Yes	Yes	Yes	Yes	Yes	Yes	1. Retrospective observational study using propensity score analyses<br>2. Operationally orientated, aiming to optimise transport protocols based on prehospital indicators. No direct use of OR/OM tools; propensity score analysis is used to balance covariates between directly and indirectly transported patients, facilitating reliable outcome comparison.
Dinh et al. [105]	Outcome	Yes	Yes	Yes	Yes	Yes	Yes	Yes	1. Retrospective cohort study using integer-valued autoregressive Poisson models and multivariable logistic regression<br>2. Operationally orientated, focusing on improving patient outcomes through quality assurance in trauma care. No direct use of OR/OM tools; statistical models evaluate the program's impact on mortality and rehabilitation, highlighting its role in enhancing trauma care quality.
Curtis et al. [63]	Process	Yes	Yes	Yes	Yes	Yes	Yes	Yes	1. Retrospective analysis using general linear models<br>2. Operationally orientated, aiming to improve resource allocation and patient outcomes through optimised triage. No direct use of OR/OM tools; statistical models evaluate the effectiveness of the tiered response system, highlighting potential improvements in trauma team activation criteria.
Sewalt et al. [154]	Outcome	Yes	Yes	Yes	Yes	Yes	Yes	Yes	1. Retrospective observational cohort study using multivariable random effects logistic regression<br>2. Operationally orientated, focusing on evaluating trauma centre performance with respect to patient volume. No direct use of OR/OM tools; statistical models are used to explore volume-outcome relationships and assess the centralisation of trauma care.
Andruszkow et al. [137]	Outcome	Yes	Yes	Yes	Yes	Yes	Yes	Yes	1. Retrospective cohort study using multivariate regression analysis<br>2. Operationally orientated, focusing on the effectiveness of Helicopter Emergency Medical Services (HEMS) in trauma care to inform rescue and triage strategies. There is no direct use of OR/OM tools; statistical models assess HEMS' impact on survival outcomes, highlighting its role in trauma system optimisation.

Prepared using sagej.cls

Table 2 continued from previous page

Napoli et al. [159]	Outcome	Yes	Yes											Yes	Yes	Yes	Yes	Yes											1. Retrospective cohort design, applying statistical analyses to compare the effectiveness of the RMM and W-Score<br>2. Operational Orientation: Operationally orientated, focusing on assessing trauma centre metrics for quality improvement. No direct use of OR/OM tools. It emphasises metric development and validation.
Braken et al. [51]	Process	Yes	Yes																Yes	Yes	Yes	Yes	Yes						1. Cross-sectional analysis using descriptive statistics and simulation models<br>2. Operationally oriented, focusing on optimizing trauma team activation protocols to improve resource allocation. The utilisation of OR/OM tools, specifically simulation models, to assess the impact of modified triage criteria on overtriage and undertriage rates, aiding in refining emergency response protocols.
Rahmani et al. [163]	Outcome	Yes	Yes																Yes	Yes	Yes	Yes	Yes						1. Cross-sectional observational study using ROC curve analysis<br>2. Operationally orientated, aiming to improve trauma triage accuracy. No direct use of OR/OM tools; the study relies on statistical evaluation to validate prognostic scoring tools in trauma care settings.
Majercik et al. [167]	Outcome	Yes	Yes											Yes	Yes	Yes	Yes	Yes											1. Retrospective cohort study using multivariable Cox regression and ROC analysis<br>2. Operationally orientated, focusing on enhancing early mortality risk assessment in trauma care. No direct use of OR/OM tools; the study applies statistical modelling to evaluate the clinical utility of a scoring tool for mortality prediction, informing trauma care.
Follin et al. [50]	Process	Yes	Yes											Yes	Yes	Yes	Yes	Yes											1. Prospective observational study, using recursive partitioning to create a decision tree model<br>2. Operationally orientated, aiming to enhance prehospital triage and resource allocation in trauma care. The utilisation of OR/OM tools, specifically a decision tree model, to improve triage accuracy and minimise overtriage and undertriage, contributing to optimal trauma system efficiency.
Truchon et al. [21]	All	Yes	Yes																Yes	Yes	Yes	Yes	Yes						1. Descriptive observational study<br>2. Operationally orientated, as it focuses on quality improvement and system optimization. Utilisation of OR/OM tools such as continuous monitoring and reporting of performance metrics to maintain and enhance trauma care standards across Quebec.
Jensen et al. [42]	Process	Yes	Yes											Yes	Yes	Yes	Yes	Yes											1. A retrospective observational study using Bayesian variable selection was conducted to identify significant pre-hospital triage predictors and cross-validation methods (Brier and logarithmic scores) for predictive accuracy.<br>2. Operationally orientated, aiming to optimise triage criteria to reduce overtriage and undertriage rates in trauma centres. Utilisation of OR/OM tools, specifically predictive modelling and Bayesian variable selection, to refine pre-hospital triage parameters, enhancing trauma system efficiency.
Osler et al. [160]	Outcome	Yes	Yes											Yes	Yes	Yes	Yes	Yes											1. Retrospective cohort study using probit regression and model, validated with ROC curves and Hosmer-Lemeshow goodness-of-fit tests.<br>2. Operationally orientated, aiming to enhance trauma risk adjustment and predictive accuracy. The use of predictive modelling and statistical calibration contributes to the trauma system's performance improvement and outcome predictions.
Rhinehart et al. [143]	Outcome	Yes	Yes											Yes	Yes	Yes	Yes	Yes											1. Retrospective cohort study using multivariable logistic regression<br>2. Operationally orientated, aiming to optimise trauma transport resources by understanding airbase distribution impacts on mortality. No direct use of OR/OM tools; statistical analysis is applied to assess the effect of geographic distribution on patient outcomes, supporting trauma network planning and airbase allocation strategies.
Girard et al. [136]	Outcome	Yes	Yes																Yes	Yes	Yes	Can't tell	Yes						1. Retrospective descriptive design<br>2. Operationally orientated, focusing on quality improvement in trauma care. Systematic error reporting and categorising were adopted to identify operational gaps in trauma management. No direct use of OR/OM tools.
Gallagher et al. [149]	Outcome	Yes	Yes											Yes	Yes	Yes	Yes	Yes											1. Retrospective cohort study using Cox regression analysis<br>2. Operationally orientated, aiming to understand the role of pretransfer imaging in trauma care outcomes. No direct use of OR/OM tools; statistical modelling is applied to evaluate imaging effects on patient survival, supporting potential refinements in transfer protocols.
Sullivent et al. [142]	Outcome	Yes	Yes											Yes	Yes	Yes	Yes	Yes											1. Retrospective cohort study using logistic regression<br>2. Operationally orientated, aiming to assess the effectiveness of HEMS in reducing mortality for trauma patients. No direct use of OR/OM tools; statistical modelling is used to evaluate the impact of transport mode on patient outcomes, supporting policy decisions for emergency transport practices.
Cudnik et al. [146]	Outcome	Yes	Yes											Yes	Yes	Yes	Yes	Yes											1. Prospective observational study using multivariable logistic regression<br>2. Operationally orientated, as it seeks to refine criteria for air transport decisions to improve outcomes and resource allocation. No direct use of OR/OM tools; the use of statistical analysis to support more effective triage criteria for HEMS transport in trauma care.

Table 2 continued from previous page

Schluter [164]	Outcome	Yes	Yes	Yes	Yes	Yes	Yes	Yes						1. Retrospective study using logistic regression and interaction terms to revise the TRISS model<br>2. Operationally orientated, aiming to enhance trauma outcome prediction for benchmarking trauma care. No direct use of OR/OM tools; uses logistic regression and interaction terms to refine TRISS model predictions for improved trauma system evaluation and performance monitoring
Ryb et al. [139]	Outcome	Yes	Yes	Yes	Yes	Yes	Yes	Yes						1. Retrospective cohort study using multivariable logistic regression<br>2. Operationally orientated, focusing on the role of HEMS in trauma care. No direct use of OR/OM tools; the study uses statistical analysis to evaluate transport mode impacts on patient survival, supporting trauma care policy decisions.
De Jongh et al. [147]	Outcome	Yes	Yes	Yes	Yes	Yes	Yes	Yes						1. Retrospective matched-pair cohort study using multivariate logistic regression<br>2. Operationally orientated, aiming to assess HEMS effectiveness in improving trauma patient outcomes. No direct use of OR/OM tools; the study uses statistical modelling to evaluate HEMS's impact on survival, supporting potential improvements in trauma care transport policies.
Madiraju et al. [66]	Process	Yes	Yes	Yes	Yes	Yes	Yes	Yes						1. Retrospective study with multivariate regression analysis<br>2. Operationally orientated, aiming to reduce overtriage costs in trauma centres. No direct use of OR/OM tools; the study uses statistical analysis to inform resource allocation for trauma transport.
Lam et al. [166]	Outcome	Yes	Yes	Yes	Yes	Yes	Yes	Yes						1. Retrospective cohort study using logistic regression to evaluate the predictive performance of the BISS model compared to TRISS and ASCOT, with performance metrics like AUC and calibration.<br>2. Operationally orientated, aiming to provide a simplified survival prediction model for trauma care evaluation. No direct use of OR/OM tools; uses statistical analysis to validate predictive models, supporting quality assessment and outcome benchmarking in trauma care.
Ang et al. [133]	Outcome	Yes	Yes	Yes	Yes	Yes	Yes	Yes						1. Retrospective study using multivariate logistic regression and patient safety indicators (PSIs)<br>2. Operationally orientated, aiming to identify and address areas for improvement in trauma care at a statewide level. No direct use of OR/OM tools; uses statistical benchmarking to assess trauma centre performance and patient safety outcomes.
Tsuchiya et al. [144]	Outcome	Yes	Yes	Yes	Yes	Yes	Yes	Yes						1. A retrospective cohort design with statistical adjustments, including propensity score matching and instrumental variable analysis.<br>2. Operationally orientated, focusing on optimising trauma care transport to improve patient outcomes, though it relies on statistical analysis rather than OR/OM tools directly.
Shaw et al. [145]	Outcome	Yes	Yes	Yes	Yes	Yes	Yes	Yes						1. Retrospective cohort study using multivariable logistic regression<br>2. Operationally orientated, aiming to evaluate the effectiveness of helicopter transport in trauma care. No direct use of OR/OM tools; statistical analysis and GIS data are used to assess the transport mode's effect on outcomes, supporting decisions in trauma transport practices.
Balvers et al. [174]	Outcome	Yes	Yes	Yes	Yes	Yes	Yes	Yes						1. Retrospective cohort study with logistic regression<br>2. Operationally orientated, focusing on improving trauma patient outcomes through predictive modelling of mortality risk based on physiological indicators. No direct use of OR/OM tools; the study leverages logistic regression for risk stratification, aiding in optimising patient management and ICU resource allocation.
Larsson et al. [78]	Process	Yes	Yes	Yes	Yes	Yes	Can't tell	Yes						1. A simulation study comparing XGBoost and logistic regression models<br>2. Operationally orientated, with a focus on enhancing trauma triage through machine learning and simulation to optimise patient routing to trauma centres. It potentially aids in resource allocation and improving patient outcomes in emergency settings.
Little et al. [150]	Outcome	Yes	Yes	Yes	Yes	Yes	Can't tell	Yes						1. Retrospective observational study<br>2. Not operationally orientated, as it primarily examines mortality outcomes without directly focusing on system optimisation or operational improvements. The study does not directly use OR/OM tools but contributes to understanding potential outcome disparities related to admission timing, which could inform resource planning indirectly.
Strosberg et al. [99]	Process	Yes	Yes	Yes	Yes	Yes	Yes	Yes						1. Retrospective study using logistic regression<br>2. The study is not operationally oriented, as its primary focus is on readmission outcomes based on discharge destinations rather than systemic or process improvements in trauma care. The study does not directly utilize OR/OM tools, yet the results could guide discharge planning and resource distribution for elderly trauma patients, potentially influencing operational choices.
Navaratne et al. [88]	Process	Yes	Yes						Yes	Yes	Yes	Yes	Yes	1. Retrospective observational study<br>2. The study is not operationally orientated, with the primary goal being to describe trends in trauma demographics and outcomes rather than directly focusing on system optimisation. No direct use of OR/OM tools.

Prepared using sagej.cls

Table 2 continued from previous page

Henriksson et al. [62]	Process	Yes	Yes	Yes	Yes	Yes	Can't tell	Yes						1. Registry-based cohort study using logistic regression models<br>2. Operationally orientated, aiming to optimise trauma care by identifying risks associated with mistriage when prediction models are applied across diverse clinical settings. No direct use of OR/OM tools; the study instead uses logistic regression models to analyse model transfer impacts, informing trauma care resource allocation and patient safety across trauma networks.
Ardolino et al. [176]	Outcome	Yes	Yes						Yes	Yes	Yes	Yes	Yes	1. Consensus-based study incorporating a systematic review<br>2. Not operationally orientated, mainly focusing on recommending standardised outcome metrics for trauma rather than on optimising or improving operational processes directly. No direct use of OR/OM tools.
Durston et al. [89]	Process	Yes	Yes						Yes	Yes	Yes	Yes	Yes	1. Retrospective study using statistical analysis<br>2. Operationally orientated, focusing on the temporal distribution of trauma cases to inform resource allocation and staffing at peak times. No direct use of OR/OM tools; the statistical analysis supports planning and operational adjustments, helping optimise staffing and resource deployment to meet predictable demand patterns in trauma care.
McHenry and Smith [40]	Structure	Yes	Yes	Yes	Yes	Can't tell	Yes	Yes						1. Retrospective cohort design with logistic regression and geospatial analysis<br>2. Operationally orientated, focusing on enhancing trauma system efficiency by informing resource deployment based on geographic and temporal factors. It employs geospatial modelling as a methodological tool to evaluate service allocation needs.
O'Reilly et al. [121]	Outcome	Yes	Yes	Yes	Yes	Yes	Yes	Yes						1. Retrospective study using logistic regression<br>2. Not operationally orientated; primarily descriptive, focusing on survival outcomes relative to hospital population density rather than on system optimisation. No direct use of OR/OM tools; results may guide trauma system planning, particularly in resource distribution across urban and rural trauma centres.
Nilsbakken et al. [123]	Outcome	Yes	Yes	Yes	Yes	Yes	Yes	Yes						1. Population-based cohort study using binary logistic regression<br>2. Operationally orientated, focusing on identifying geographical and time-based factors affecting trauma outcomes with potential implications for emergency response optimisation. No direct use of OR/OM tools; logistic regression provides insights to improve trauma response planning and resource allocation in varied geographic settings.
Dehli et al. [122]	Outcome	Yes	Yes	Yes	Yes	Yes	Yes	Yes						1. Retrospective cohort study using logistic regression<br>2. Operationally orientated, aiming to identify disparities in trauma outcomes to inform improvements in trauma care delivery and hospital referral practices. No direct use of OR/OM tools; however, statistical modelling offers insights that could guide trauma resource allocation and transport planning in remote regions.
Kwon et al. [118]	Outcome	Yes	Yes	Yes	Yes	Yes	Yes	Yes						1. Retrospective cohort study<br>2. Operationally orientated, aiming to enhance trauma outcomes by implementing a structured national trauma system with designated centres and improved transfer protocols. No direct use of OR/OM tools; however, findings inform system-level policy and resource allocation strategies for trauma care improvement.
Cohen et al. [76]	Process	Yes	Yes	Yes	Yes	Yes	Yes	Yes						1. Retrospective cohort study using logistic regression<br>2. Operationally orientated, focusing on improving trauma triage processes to optimise patient outcomes and resource allocation. No direct use of OR/OM tools; logistic regression provides insights that may support adjustments to triage protocols, potentially informing efficient resource utilisation in trauma centres.
Hirpara et al. [30]	Structure	Yes	Yes	Yes	Yes	Yes	Yes	Yes						1. A quantitative study using a nested multi-level optimisation model<br>2. Operationally orientated, aiming to optimise trauma network design and enhance resource allocation for improved patient safety outcomes. The multi-objective optimisation model guides the trauma centre placement and resource distribution, supporting policy decisions in trauma care systems.
Amato et al. [126]	Outcome	Yes	Yes	Yes	Yes	Yes	Yes	Yes						1. Ecological study using GIS and geographically weighted regression (GWR)<br>2. Operationally orientated, informing trauma system planning by analysing spatial access and healthcare outcomes. No direct use of OR/OM tools; GIS and GWR provide insights that may support trauma centre designation and resource allocation to improve population health outcomes.
Stonko et al. [102]	Outcome	Yes	Yes	Yes	Yes	Yes	Yes	Yes						1. Pilot study using ANN for the high length of stay prediction<br>2. Operationally orientated, aiming to optimise resource allocation by predicting high LOS. No direct use of OR/OM tools; ANN modelling provides insights that may support early intervention and efficient resource distribution in trauma care settings.
Parikh et al. [32]	Structure	Yes	Yes	Yes	Yes	Can't tell	Yes	Yes						1. Retrospective observational design with optimisation modelling.<br>2. Operationally orientated, focusing on improving trauma system efficiency and resource allocation using an optimisation-based approach to inform policy decisions on trauma centre distribution.

Table 2 continued from previous page

Tiruneh et al. [151]	Outcome	Yes	Yes											Yes	Yes	Yes	Yes	Yes											1. Retrospective cohort study using logistic regression<br>2. Operationally orientated, aiming to improve trauma outcomes by assessing admission pathways to guide triage and transfer policies. No direct use of OR/OM tools; statistical analysis informs recommendations for optimal trauma centre admissions to improve patient survival.
Beck et al. [31]	Structure	Yes	Yes											Yes	Yes	Yes	Yes	Yes											1. Geospatial analysis with a Mixed Integer Linear Programming (MILP) model<br>2. Operationally orientated, aiming to optimise trauma network configurations and enhance resource allocation. The MILP model provides evidence-based insights to support trauma system planning and improve patient access across various geographic scenarios.
Waalwijk et al. [117]	Outcome	Yes	Yes											Yes	Yes	Yes	Yes	Yes											1. Observational cohort study using generalised linear models with inverse probability weighting<br>2. Operationally orientated, focusing on optimising trauma triage and transfer practices to enhance patient survival. No direct use of OR/OM tools; statistical analysis informs transfer protocol improvements to support optimal patient outcomes in trauma systems.
Plurad et al. [134]	Outcome	Yes	Yes											Yes	Yes	Yes	Yes	Yes											1. Retrospective cohort study using logistic regression<br>2. Not operationally orientated. The study focuses on outcome comparison rather than system optimisation or process improvement. No direct use of OR/OM tools; however, findings may guide policy regarding trauma centre verification and inform healthcare resource planning across trauma care systems.
Lin et al. [96]	Process	Yes	Yes											Yes	Yes	Yes	Can't tell	Yes											1. Discrete choice modelling (DCM)<br>2. Operationally orientated, focusing on examining decision factors for trauma transfers that can inform improvements in transfer criteria and processes. DCM is used to model decision-making, supporting policy design for trauma transfer protocols.
Shi et al. [75]	Process	Yes	Yes											Yes	Yes	Yes	Yes	Yes											1. Retrospective cohort study using hierarchical logistic regression<br>2. Operationally orientated, aiming to improve trauma outcomes by examining the role of funding and re-triage. No direct use of OR/OM tools; hierarchical logistic regression supports trauma system planning and resource allocation decisions by identifying funding and triage practices linked to mortality reduction.
Trier et al. [119]	Outcome	Yes	Yes																Yes	Yes	Yes	Yes	Yes						1. Retrospective cohort study using logistic regression<br>2. Not operationally orientated; primarily descriptive, focusing on trends in patient age, injury mechanism, and outcomes. No direct use of OR/OM tools; findings may support trauma care planning and resource allocation for older patients and fall-related injuries.
Reitano et al. [171]	Outcome	Yes	Yes																Yes	Yes	Yes	Yes	Yes						1. Retrospective using multivariate logistic regression<br>2. Not operationally orientated, as the focus is on understanding mortality trends rather than on optimising or improving operational processes directly. While OR/OM tools are not directly utilised, the results may guide clinical protocols and resource planning, especially in acute trauma care, by emphasising risk factors linked to various forms of trauma mortality.
Van Den Driessche et al. [124]	Outcome	Yes	Yes																Yes	Yes	Yes	Yes	Yes						1. Retrospective cohort study using logistic regression<br>2. Operationally orientated, aiming to inform trauma triage and improve patient outcomes through understanding admission and transfer patterns. No direct use of OR/OM tools; statistical analysis supports operational insights for refining triage protocols and optimising patient allocation within trauma networks.
Van Ditshuizen et al. [181]	Outcome	Yes	Yes																Yes	Yes	Yes	Can't tell	Yes						1. Retrospective cohort study<br>2. Not operationally orientated, as the study focuses on describing HRQoL and RTW outcomes rather than on optimising trauma care systems or resource use. No direct use of OR/OM tools; however, the findings may inform clinical practices and support planning for long-term rehabilitation resources.
Hung et al. [180]	Outcome	Yes	Yes																Yes	Yes	Yes	Can't tell	Yes						1. Prospective cohort study<br>2. Not operationally orientated, as the focus is on evaluating patient outcomes rather than optimising trauma care systems. No direct use of OR/OM tools; the study is primarily descriptive, though findings may indirectly inform resource planning and rehabilitation services for long-term trauma care.
Cho et al. [27]	Structure	Yes	Yes											Yes	Yes	Yes	Yes	Yes											1. Optimisation study applying a mixed-integer nonlinear programming (MINLP) model<br>2. Operationally Operationally orientated, focusing on enhancing trauma system efficiency by optimising facility and resource locations. The customised MINLP and simulation support EMS planning, aligning closely with operations research objectives in healthcare logistics and resource allocation.
Bélanger et al. [29]	Structure	Yes	Yes											Yes	Yes	Yes	Yes	Yes											1. Discrete event simulation (DES) integrated with mathematical optimisation<br>2. Operationally orientated, using a simulation-optimisation approach to dynamically update system parameters based on ongoing operations data, enhancing real-time decision-making capabilities in EMS systems.

Prepared using sagej.cls

Table 2 continued from previous page

Kunene and Weistroffer [33]	Structure	Yes	Yes											Yes	Yes	Yes	Yes	Can't tell	1. Predictive modelling using MCDA and decision rules<br>2. Operationally oriented within healthcare; employs decision tree induction, MCDA.
Hyer et al. [34]	Structure	Yes	Yes											Yes	Yes	Yes	Yes	Yes	1. Case study with quantitative analysis of pre-and post-implementation metrics in a focused trauma unit<br>2. Operationally orientated, aiming to improve hospital trauma care efficiency by introducing a focused unit. No direct use of OR/OM tools; the study primarily relies on quantitative performance analysis to assess operational improvements, which aligns with operations management principles for evaluating process redesign and resource utilisation in healthcare settings.
Faraj and Xiao [35]	Structure	Yes	Yes	Yes	Yes	Yes	Yes	Yes											1. An ethnographic study utilising observations, interviews, and thematic analysis<br>2. Operationally orientated, focusing on examining how trauma centres manage uncertainty and task interdependence to ensure effective patient care. No direct use of OR/OM tools; the study instead uses qualitative analysis to explore the coordination of resources and expertise in real-time decision-making, contributing insights relevant to operations management by examining resource flexibility and interdependence in healthcare contexts.
Anderson et al. [36]	Structure	Yes	Yes											Yes	Yes	Yes	Yes	Yes	1. Retrospective study using regression models<br>2. Operationally oriented, aiming to improve hospital operations by identifying resource-related discrepancies in trauma care quality based on arrival times. No direct use of OR/OM tools; the study employs regression analysis to explore within-hospital quality variation, focusing on implications for staffing and resource allocation, which align with the objectives of operations management in healthcare.
Allon et al. [39]	Structure	Yes	Yes											Yes	Yes	Yes	Yes	Yes	1. Cross-sectional study using queueing theory and empirical modelling<br>2. Operationally orientated, focusing on improving hospital and ED operations through capacity management insights. The utilisation of queueing theory and simulation was applied to explore solutions for reducing diversion and enhancing ED efficiency, aligning with goals in operations management for healthcare systems.
Webb and Mills [38]	Structure	Yes	Yes											Yes	Yes	Yes	Can't tell	Yes	1. Theoretical theoretical modelling for decision analysis<br>2. Operationally orientated, aiming to optimise EMS and hospital operations by identifying economically viable triage policies that could reduce emergency department congestion. The study uses decision modelling to explore system improvements, focusing on EMS efficiency and resource allocation in healthcare.

Table 3. Summary of Critical Appraisal Skills Program (CASP) results for identified systematic review studies

	Hill et al.[152]	Aringhieri et al.[37]	Morris et al.[54]	Celso et al.[103]	Ahmadi-Javid et al.[28]	Jones et al.[182]	Van Ditshuizen et al.[125]
1. Did the systematic review address a clearly formulated research question?	Yes	Yes	Yes	Yes	Yes	Yes	Yes
2. Did the researchers search for appropriate study designs to answer the research question?	Yes	Yes	Yes	Yes	Yes	Yes	Yes
3. Were all relevant primary research studies likely to have been included in the systematic review?	Yes	Can't tell	Can't tell	Can't tell	Can't tell	Can't tell	Yes
4. Did the researchers assess the validity or methodological rigor of the primary research studies included in the systematic review?	Yes	No	No	Yes	No	Yes	Yes
5. Did the researchers extract and present information on the individual primary research studies appropriately and transparently?	Yes	Yes	Yes	Yes	Yes	Yes	Yes
6. Did the researchers analyze the results of the individual primary research studies appropriately?	Yes	Yes	Yes	Yes	Yes	Yes	Yes
7. Did the researchers report any limitations of the systematic review and, if so, do the limitations discussed cover all the issues in your critical appraisal?	Yes	No	No	Yes	No	Yes	Yes
8. Would the benefits of acting upon the results outweigh any potential disadvantages, harms, and/or additional demand for resources associated with acting on the results?	Can't tell	Can't tell	Can't tell	Yes	Can't tell	Can't tell	Can't tell
9. Can the results of the systematic review be applied to your local population/in your local setting or context?	Yes	Yes	Yes	Yes	Yes	Yes	Yes
10. If actioned, would the findings from the systematic review represent greater or additional value for the individuals or populations for whom you are responsible?	Can't tell	Can't tell	Can't tell	Yes	Can't tell	Can't tell	Can't tell

Prepared using sagej.cls

Prepared using sagej.cls

Table 4

Study characteristics	Number	Reference
**Country**		
USA	67	Crowley et al.[71], Sborov et al.[141], Morgan et al.[82], Holena et al.[115], Vernon et al.[114], Dennis et al.[101], Adzemovic et al.[74], Polites et al.[55], Miller et al.[168], Boyd et al.[120], Brown et al.[172], Ciesla et al.[58], He et al.[113], Ashley et al.[112], Shawhan et al.[49], Ciesla et al.[43], Minei et al.[155], Brown et al.[138], Newgard et al.[44], Brown et al.[65], Osen et al.[69], Nirula et al.[73], Barringer et al.[94], Esposito et al.[93], Sewalt et al.[64], Deeb et al.[59], Karrison et al.[148], Jammula et al.[53], Stonko et al.[100], Byrne et al.[156], Heaney et al.[132], Horst et al.[56], Chen et al.[68], Shafi et al.[135], Guyette et al.[169], Haas et al.[131], Rivara et al.[72], Faul et al.[95], Vachon et al.[98], Calland and Stukenborg[153], Haider et al.[52], Nirula et al.[73], Garwe et al.[60], Napoli et al.[159], Majercik et al.[167], Osler et al.[160], Rhinehart et al.[143], Gallagher et al.[149], Sullivent et al.[142], Cudnik et al.[146], Ryb et al.[139], Madiraju et al.[66], Ang et al.[133], Shaw et al.[145], Strosberg et al.[99], Hirpara et al.[30], Amato et al.[126], Stonko et al.[102], Parikh et al.[32], Plurad et al.[134], Shi et al.[75], Kunene and Weistroffer[33], Hyer et al.[34], Faraj and Xiao[35], Anderson et al.[36], Allon et al.[39], Webb and Mills[38]
Canada	19	Tillmann et al.[70], Moore et al.[80], Moore et al.[110], Moore et al.[20], McKee et al.[108], Kuimi et al.[87], Kuimi et al.[81], Scerbo et al.[77], Moore et al.[79], Moore et al.[97], Moore et al.[18], Moore et al.[19], Moore et al.[17], Gomez et al.[91], Moore et al.[111], Hameed et al.[109], Truchon et al.[21], Lin et al.[96], Bélanger et al.[29]
England and Wales	16	Hewitt et al.[92], Haslam et al.[83], Beaumont et al.[140], Moran et al.[86], Thompson et al.[170], Metcalfe et al.[85], Kieffer et al.[90], Metcalfe et al.[177], Davenport et al.[128], Wohlgemut et al.[57], Sewalt et al.[154], Little et al.[150], Navaratne et al.[88], Ardolino et al.[176], Durston et al.[89], McHenry and Smith[40]
Netherland	14	Dinh et al.[173], Cameron et al.[107], Gomez et al.[106], Wong et al.[127], Taylor et al.[67], Dinh et al.[105], Curtis et al.[63], Ben Beck et al.[31]
Australia	8	Dinh et al.[173], Cameron et al.[107], Gomez et al.[106], Wong et al.[127], Taylor et al.[67], Dinh et al.[105], Curtis et al.[63], Ben Beck et al.[31]
multiple countries	9	van Rein et al.[48], Cameron et al.[41], Celso et al.[103], Morris et al.[54], Hill et al.[152], Van Ditshuizen et al.[125], Jones et al.[182], Ahmadi-Javid et al.[28], Aringhieri et al.[37]
Germany	4	Ruchholtz et al.[129], Ruchholtz et al.[130], Andruszkow et al.[137], Braken et al.[51]
Italy	4	Martino et al.[178], Fugazzola et al.[161], Magnone et al.[187], Reitano et al.[171]
France	3	Sartorius et al.[162], Follin et al.[50], Girard et al.[136]
Switerland	3	Jensen et al.[42], Larsson et al.[78], Henriksson et al.[62]
Others	13	Jansen et al.[116], Rahmani et al.[163], Schluter[164], Tsuchiya et al.[144], O'Reilly et al.[121], Nilsbakken et al.[123], Dehli et al.[122], Kwon et al.[118], Cohen et al.[76], Tiruneh et al.[151], Trier et al.[119], Hung et al.[180]
**Study emphasis**		

Operational	88	McHenry and Smith[40], Hirpara et al.[30], Parikh et al.[32], Ben Beck et al.[31], Cho et al.[27], Ahmadi-Javid et al.[28], Bélanger et al.[29], Kunene and Weistroffer[33], Hyer et al.[34], Faraj and Xiao[35], Anderson et al.[36], Allon et al.[39], Webb and Mills[38], Aringhieri et al.[37], Crowley et al.[71], Hewitt et al.[92], Haslam et al.[83], Morgan et al.[82], Tillmann et al.[70], Havermans et al.[84], Dennis et al.[101], Adzemovic et al.[74], Moran et al.[86], Moore et al.[80], Polites et al.[55], Voskens et al.[45], Ciesla et al.[58], van Rein et al.[48], Metcalfe et al.[85], Kieffer et al.[90], Kuimi et al.[87], Kuimi et al.[81], Shawhan et al.[49], Ciesla et al.[43], Scerbo et al.[77], Cameron et al.[41], Moore et al.[79], Moore et al.[97], Gomez et al.[91], Newgard et al.[44], Brown et al.[65], Osen et al.[69], Nirula et al.[73], Ocak et al.[47], Barringer et al.[94], Sturms et al.[46], Esposito et al.[93], Morris et al.[54], Sewalt et al.[64], Wohlgemut et al.[57], Deeb et al.[59], Jammula et al.[53], Stonko et al.[100], Horst et al.[56], Chen et al.[68], Taylor et al.[67], Rivara et al.[72], Magnone et al.[187], Sturms et al.[61], Faul et al.[95], Vachon et al.[98], Haider et al.[52], Nirula et al.[73], Garwe et al.[60], Curtis et al.[63], Braken et al.[51], Follin et al.[50], Jensen et al.[42], Madiraju et al.[66], Larsson et al.[78], Strosberg et al.[99], Navaratne et al.[88], Henriksson et al.[62], Durston et al.[89], Cohen et al.[76], Lin et al.[96], Shi et al.[75], Ardolino et al.[176], Metcalfe et al.[177], Gunning et al.[179], Van Den Driessche et al.[181], Hung et al.[180], Jones et al.[182], Moore et al.[20], Moore et al.[18], Moore et al.[19], Moore et al.[17], Truchon et al.[21], Nirula and Brasel[175]
Clincal	72	Martino et al.[178], Beaumont et al.[140], Fugazzola et al.[161], Sborov et al.[141], Dinh et al.[173], Cameron et al.[107], Holena et al.[115], Vernon et al.[114], Gomez et al.[106], Miller et al.[168], Thompson et al.[170], Moore et al.[110], Boyd et al.[120], Brown et al.[172], He et al.[113], Jansen et al.[116], McKee et al.[108], Ashley et al.[112], Ruchholtz et al.[129], Gunning and Leenen[165], Minei et al.[155], Brown et al.[138], Davenport et al.[128], Moore et al.[111], Hameed et al.[109], Celso et al.[103], Berkeveld et al.[157], Karrison et al.[148], Byrne et al.[156], Heaney et al.[132], Ruchholtz et al.[130], Wong et al.[127], Shafi et al.[135], Hill et al.[152], Guyette et al.[169], Haas et al.[131], Sartorius et al.[162], Calland and Stukenborg[153], Dinh et al.[105], Sewalt et al.[154], Andruszkow et al.[137], Napoli et al.[159], Rahmani et al.[163], Majercik et al.[167], Osler et al.[160], Rhinehart et al.[143], Girard et al.[136], Gallagher et al.[149], Sullivent et al.[142], Cudnik et al.[146], Schluter[164], Ryb et al.[139], de Jongh et al.[147], Lam et al.[166], Ang et al.[133], Tsuchiya et al.[144], Shaw et al.[145], Balvers et al.[174], Little et al.[150], O'Reilly et al.[121], Nilsbakken et al.[123], Dehli et al.[122], Kwon et al.[118], Van Ditshuizen et al.[125], Amato et al.[126], Stonko et al.[102], Tiruneh et al.[151], Van Waalwijk et al.[117], Plurad et al.[134], Trier et al.[119], Reitano et al.[171], Van Ditshuizen et al.[124]
**Research scope**		

Prepared using sagej.cls

single centre	44	Crowley et al.[71], Martino et al.[178], Sborov et al.[141], Havermans et al.[84], Gunning et al.[179], Shawhan et al.[49], Scerbo et al.[77], Davenport et al.[128], Ocak et al.[47], Barringer et al.[94], Berkeveld et al.[157], Wohlgemut et al.[57], Stonko et al.[100], Heaney et al.[132], Wong et al.[127], Shafi et al.[135], Guyette et al.[169], Rivara et al.[72], Magnone et al.[187], Vachon et al.[98], Dinh et al.[105], Curtis et al.[63], Napoli et al.[159], Rahmani et al.[163], Majercik et al.[167], Follin et al.[50], Jensen et al.[42], Gallagher et al.[149], de Jongh et al.[147], Madiraju et al.[66], Lam et al.[166], Shaw et al.[145], Little et al.[150], Strosberg et al.[99], Navaratne et al.[88], Durston et al.[89], McHenry and Smith[40], Cohen et al.[76], Stonko et al.[102], Trier et al.[119], Reitano et al.[171], Kunene and Weistroffer[33], Hyer et al.[34], Faraj and Xiao[35]
regional	55	Hewitt et al.[92], Haslam et al.[83], Beaumont et al.[140], Fugazzola et al.[161], Morgan et al.[82], Tillmann et al.[70], Dinh et al.[173], Holena et al.[115], Vernon et al.[114], Moran et al.[86], Gomez et al.[106], Voskens et al.[45], Thompson et al.[170], Ciesla et al.[58], He et al.[113], Metcalfe et al.[85], Jansen et al.[116], Moore et al.[20], McKee et al.[108], Ashley et al.[112], Kuimi et al.[87], Kuimi et al.[81], Metcalfe et al.[177], Ciesla et al.[43], Gunning and Leenen[165], Moore et al.[97], Moore et al.[18], Moore et al.[19], Moore et al.[17], Gomez et al.[91], Moore et al.[111], Sturms et al.[46], Esposito et al.[93], Deeb et al.[59], Karrison et al.[148], Jammula et al.[53], Horst et al.[56], Chen et al.[68], Taylor et al.[67], Garwe et al.[60], Truchon et al.[21], Rhinehart et al.[143], Girard et al.[136], Cudnik et al.[146], Ang et al.[133], Balvers et al.[174], Parikh et al.[32], Ben Beck et al.[31], Van Waalwijk et al.[117], Plurad et al.[134], Lin et al.[96], Van Ditshuizen et al.[124], Van Den Driessche et al.[181], Hung et al.[180], Bélanger et al.[29]
cross-regional	13	Dennis et al.[101], Kieffer et al.[90], Minei et al.[155], Brown et al.[138], Newgard et al.[44], Haas et al.[131], Sartorius et al.[162], Sullivent et al.[142], Schluter[164], Larsson et al.[78], Hirpara et al.[30], Shi et al.[75], Allon et al.[39]
national	39	Cameron et al.[107], Adzemovic et al.[74], Moore et al.[80], Polites et al.[55], Miller et al.[168], Moore et al.[110], Boyd et al.[120], Brown et al.[172], Ruchholtz et al.[129], Moore et al.[79], Hameed et al.[109], Brown et al.[65], Osen et al.[69], Nirula et al.[73], Sewalt et al.[64], Byrne et al.[156], Ruchholtz et al.[130], Sturms et al.[61], Faul et al.[95], Calland and Stukenborg[153], Haider et al.[52], Nirula et al.[73], Sewalt et al.[154], Andruszkow et al.[137], Braken et al.[51], Osler et al.[160], Ryb et al.[139], Tsuchiya et al.[144], Henriksson et al.[62], Ardolino et al.[176], O'Reilly et al.[121], Nilsbakken et al.[123], Dehli et al.[122], Kwon et al.[118], Amato et al.[126], Tiruneh et al.[151], Cho et al.[27], Anderson et al.[36], Webb and Mills[38]
worldwide	9	van Rein et al.[48], Cameron et al.[41], Celso et al.[103], Morris et al.[54], Hill et al.[152], Van Ditshuizen et al.[125], Jones et al.[182], Ahmadi-Javid et al.[28], Aringhieri et al.[37]
Stucture	14	McHenry and Smith[40], Hirpara et al.[30], Parikh et al.[32], Ben Beck et al.[31], Cho et al.[27], Ahmadi-Javid et al.[28], Bélanger et al.[29], Kunene and Weistroffer[33], Hyer et al.[34], Faraj and Xiao[35], Anderson et al.[36], Allon et al.[39], Webb and Mills[38], Aringhieri et al.[37]

Process	63	Crowley et al.[71], Hewitt et al.[92], Haslam et al.[83], Morgan et al.[82], Tillmann et al.[70], Havermans et al.[84], Dennis et al.[101], Adzemovic et al.[74], Moran et al.[86], Moore et al.[80], Polites et al.[55], Voskens et al.[45], Ciesla et al.[58], van Rein et al.[48], Metcalfe et al.[85], Kieffer et al.[90], Kuimi et al.[87], Kuimi et al.[81], Shawhan et al.[49], Ciesla et al.[43], Scerbo et al.[77], Cameron et al.[41], Moore et al.[79], Moore et al.[97], Gomez et al.[91], Newgard et al.[44], Brown et al.[65], Osen et al.[69], Nirula et al.[73], Ocak et al.[47], Barringer et al.[94], Sturms et al.[46], Esposito et al.[93], Morris et al.[54], Sewalt et al.[64], Wohlgemut et al.[57], Deeb et al.[59], Jammula et al.[53], Stonko et al.[100], Horst et al.[56], Chen et al.[68], Taylor et al.[67], Rivara et al.[72], Magnone et al.[187], Sturms et al.[61], Faul et al.[95], Vachon et al.[98], Haider et al.[52], Nirula et al.[73], Garwe et al.[60], Curtis et al.[63], Braken et al.[51], Follin et al.[50], Jensen et al.[42], Madiraju et al.[66], Larsson et al.[78], Strosberg et al.[99], Navaratne et al.[88], Henriksson et al.[62], Durston et al.[89], Cohen et al.[76], Lin et al.[96], Shi et al.[75]
Outcome	78	Martino et al.[178], Beaumont et al.[140], Fugazzola et al.[161], Sborov et al.[141], Dinh et al.[173], Cameron et al.[107], Holena et al.[115], Vernon et al.[114], Gomez et al.[106], Miller et al.[168], Thompson et al.[170], Moore et al.[110], Boyd et al.[120], Brown et al.[172], Gunning et al.[179], He et al.[113], Jansen et al.[116], McKee et al.[108], Ashley et al.[112], Ruchholtz et al.[129], Metcalfe et al.[177], Gunning and Leenen[165], Minei et al.[155], Brown et al.[138], Davenport et al.[128], Moore et al.[111], Hameed et al.[109], Celso et al.[103], Berkeveld et al.[157], Karrison et al.[148], Byrne et al.[156], Heaney et al.[132], Ruchholtz et al.[130], Wong et al.[127], Shafi et al.[135], Hill et al.[152], Guyette et al.[169], Haas et al.[131], Sartorius et al.[162], Calland and Stukenborg[153], Dinh et al.[105], Sewalt et al.[154], Andruszkow et al.[137], Napoli et al.[159], Rahmani et al.[163], Majercik et al.[167], Osler et al.[160], Rhinehart et al.[143], Girard et al.[136], Gallagher et al.[149], Sullivent et al.[142], Cudnik et al.[146], Schluter[164], Ryb et al.[139], de Jongh et al.[147], Lam et al.[166], Ang et al.[133], Tsuchiya et al.[144], Shaw et al.[145], Balvers et al.[174], Little et al.[150], Ardolino et al.[176], O'Reilly et al.[121], Nilsbakken et al.[123], Dehli et al.[122], Kwon et al.[118], Van Ditshuizen et al.[125], Amato et al.[126], Stonko et al.[102], Tiruneh et al.[151], Jones et al.[182], Van Waalwijk et al.[117], Plurad et al.[134], Trier et al.[119], Reitano et al.[171], Van Ditshuizen et al.[124], Van Den Driessche et al.[181], Hung et al.[180]
All covered	5	Moore et al.[20], Moore et al.[18], Moore et al.[19], Moore et al.[17], Truchon et al.[21]
**Sample size**		
<1000	17	Martino et al.[178], Sborov et al.[141], Shawhan et al.[49], Davenport et al.[128], Sturms et al.[46], Berkeveld et al.[157], Heaney et al.[132], Taylor et al.[67], Rahmani et al.[163], Cudnik et al.[146], de Jongh et al.[147], Balvers et al.[174], Strosberg et al.[99], Navaratne et al.[88], Van Den Driessche et al.[181], Hung et al.[180], Kunene and Weistroffer[33]

Prepared using sagej.cls

1000-5000	35	Crowley et al.[71], Hewitt et al.[92], Fugazzola et al.[161], Havermans et al.[84], Voskens et al.[45], Thompson et al.[170], Gunning et al.[179], Kieffer et al.[90], Jansen et al.[116], Metcalfe et al.[177], Scerbo et al.[77], Minei et al.[155], Nirula et al.[73], Ocak et al.[47], Barringer et al.[94], Wohlgemut et al.[57], Wong et al.[127], Guyette et al.[169], Sartorius et al.[162], Magnone et al.[187], Vachon et al.[98], Garwe et al.[60], Dinh et al.[105], Braken et al.[51], Follin et al.[50], Jensen et al.[42], Gallagher et al.[149], Madiraju et al.[66], Lam et al.[166], Shaw et al.[145], Little et al.[150], Durston et al.[89], McHenry and Smith[40], Cohen et al.[76], Stonko et al.[102]
5000-10000	11	Cameron et al.[107], Holena et al.[115], Vernon et al.[114], Shafi et al.[135], Rivara et al.[72], Curtis et al.[63], Majercik et al.[167], Girard et al.[136], Ben Beck et al.[31], Trier et al.[119], Reitano et al.[171]
10000-50000	24	Dinh et al.[173], Dennis et al.[101], Gomez et al.[106], Metcalfe et al.[85], McKee et al.[108], Kuimi et al.[81], Gunning and Leenen[165], Moore et al.[17], Gomez et al.[91], Esposito et al.[93], Karrison et al.[148], Stonko et al.[100], Ruchholtz et al.[130], Sewalt et al.[154], Andruszkow et al.[137], Napoli et al.[159], Tsuchiya et al.[144], Henriksson et al.[62], O'Reilly et al.[121], Nilsbakken et al.[123], Dehli et al.[122], Tiruneh et al.[151], Van Ditshuizen et al.[124], Cho et al.[27]
50000-100000	14	Haslam et al.[83], Moore et al.[80], Moore et al.[110], Moore et al.[20], Moore et al.[97], Moore et al.[19], Brown et al.[138], Newgard et al.[44], Moore et al.[111], Osen et al.[69], Haas et al.[131], Sullivent et al.[142], Ang et al.[133], Plurad et al.[134]
>100000	35	Morgan et al.[82], Tillmann et al.[70], Adzemovic et al.[74], Moran et al.[86], Polites et al.[55], Miller et al.[168], Boyd et al.[120], Brown et al.[172], He et al.[113], Ashley et al.[112], Kuimi et al.[87], Ciesla et al.[43], Moore et al.[79], Brown et al.[65], Sewalt et al.[64], Deeb et al.[59], Jammula et al.[53], Byrne et al.[156], Horst et al.[56], Chen et al.[68], Sturms et al.[61], Faul et al.[95], Calland and Stukenborg[153], Haider et al.[52], Nirula et al.[73], Osler et al.[160], Rhinehart et al.[143], Schluter[164], Ryb et al.[139], Larsson et al.[78], Kwon et al.[118], Van Waalwijk et al.[117], Shi et al.[75], Anderson et al.[36], Webb and Mills[38]
not specified	24	Beaumont et al.[140], Ciesla et al.[58], van Rein et al.[48], Ruchholtz et al.[129], Cameron et al.[41], Moore et al.[18], Hameed et al.[109], Celso et al.[103], Morris et al.[54], Hill et al.[152], Truchon et al.[21], Ardolino et al.[176], Hirpara et al.[30], Van Ditshuizen et al.[125], Amato et al.[126], Parikh et al.[32], Jones et al.[182], Lin et al.[96], Ahmadi-Javid et al.[28], Bélanger et al.[29], Hyer et al.[34], Faraj and Xiao[35], Allon et al.[39], Aringhieri et al.[37]
**Research time scale**		
<1 year	3	Thompson et al.[170], Metcalfe et al.[177], Rahmani et al.[163]

1-5 years	97	For ¡1 year (3): Thompson et al.[170], Metcalfe et al.[177], Rahmani et al.[163] For 1-5 years (97): Crowley et al.[71], Hewitt et al.[92], Haslam et al.[83], Beaumont et al.[140], Fugazzola et al.[161], Sborov et al.[141], Dinh et al.[173], Cameron et al.[107], Vernon et al.[114], Havermans et al.[84], Dennis et al.[101], Polites et al.[55], Gomez et al.[106], Voskens et al.[45], Miller et al.[168], Boyd et al.[120], Ciesla et al.[58], Gunning et al.[179], Metcalfe et al.[85], Kieffer et al.[90], Moore et al.[20], Ashley et al.[112], Kuimi et al.[87], Kuimi et al.[81], Shawhan et al.[49], Ciesla et al.[43], Scerbo et al.[77], Moore et al.[97], Brown et al.[138], Newgard et al.[44], Davenport et al.[128], Hameed et al.[109], Brown et al.[65], Osen et al.[69], Nirula et al.[73], Ocak et al.[47], Sturms et al.[46], Esposito et al.[93], Sewalt et al.[64], Berkeveld et al.[157], Wohlgemut et al.[57], Stonko et al.[100], Byrne et al.[156], Heaney et al.[132], Ruchholtz et al.[130], Taylor et al.[67], Shafi et al.[135], Guyette et al.[169], Haas et al.[131], Sartorius et al.[162], Rivara et al.[72], Magnone et al.[187], Sturms et al.[61], Faul et al.[95], Vachon et al.[98], Calland and Stukenborg[153], Haider et al.[52], Garwe et al.[60], Dinh et al.[105], Curtis et al.[63], Sewalt et al.[154], Braken et al.[51], Follin et al.[50], Jensen et al.[42], Osler et al.[160], Gallagher et al.[149], Sullivent et al.[142], Cudnik et al.[146], Schluter[164], Ryb et al.[139], de Jongh et al.[147], Madiraju et al.[66], Lam et al.[166], Ang et al.[133], Balvers et al.[174], Little et al.[150], Strosberg et al.[99], Navaratne et al.[88], Henriksson et al.[62], Durston et al.[89], O'Reilly et al.[121], Nilsbakken et al.[123], Dehli et al.[122], Kwon et al.[118], Cohen et al.[76], Stonko et al.[102], Parikh et al.[32], Ben Beck et al.[31], Van Waalwijk et al.[117], Plurad et al.[134], Shi et al.[75], Van Ditshuizen et al.[124], Van Den Driessche et al.[181], Cho et al.[27], Hyer et al.[34], Faraj and Xiao[35], Anderson et al.[36]
6-10 years	35	Martino et al.[178], Tillmann et al.[70], Holena et al.[115], Adzemovic et al.[74], Moran et al.[86], Moore et al.[80], Moore et al.[110], He et al.[113], Jansen et al.[116], McKee et al.[108], Gunning and Leenen[165], Moore et al.[18], Moore et al.[19], Moore et al.[17], Gomez et al.[91], Moore et al.[111], Barringer et al.[94], Karrison et al.[148], Wong et al.[127], Nirula et al.[73], Andruszkow et al.[137], Majercik et al.[167], Truchon et al.[21], Rhinehart et al.[143], Girard et al.[136], Tsuchiya et al.[144], Larsson et al.[78], McHenry and Smith[40], Tiruneh et al.[151], Trier et al.[119], Reitano et al.[171], Hung et al.[180], Allon et al.[39]
11-15 years	8	Morgan et al.[82], Brown et al.[172], Moore et al.[79], Jammula et al.[53], Horst et al.[56], Chen et al.[68], Shaw et al.[145], Amato et al.[126]
>15 years	1	Deeb et al.[59]
not specified	18	van Rein et al.[48], Ruchholtz et al.[129], Cameron et al.[41], Minei et al.[155], Celso et al.[103], Morris et al.[54], Hill et al.[152], Napoli et al.[159], Ardolino et al.[176], Hirpara et al.[30], Van Ditshuizen et al.[125], Jones et al.[182], Lin et al.[96], Ahmadi-Javid et al.[28], Bélanger et al.[29], Kunene and Weistroffer[33], Webb and Mills[38], Aringhieri et al.[37]

Prepared using sagej.cls

Prepared using sagej.cls

Table 5. Summary of triage studies

References	Research Scope	Evaluation Metrics	Study Design	Analytic Methods
Crowley et al.[71]	single centre	pattern of secondary overtriage	retrospective data analysis	bivariate and multivariate logistic regression analysis
Tillmann et al.[70]	regional	the accuracy of secondary triage	retrospective data analysis	descriptive statistics and negative binomial models
Polites et al.[55]	national	undertriage rate	retrospective data analysis	descriptive statistics and multivariate logistic regression model
Voskens et al.[45]	regional	undertriage and overtriage rates	retrospective data analysis	descriptive statistics
Miller et al.[168]	national-wide	modified Rapid Emergency Medicine Score (mREMS) for trauma triage	trauma score modelling and validation study	descriptive statistics and statistical validation
Ciesla et al.[58]	regional	undertriage and overtriage rates	retrospective data analysis	descriptive statistics
van Rein et al.[48]	world-wide	prehospital triage protocols	systematic literature review	meta-analysis and quality assessment
Shawhan et al.[49]	single centre	undertriage rate,overtriage rate and mistriage rate	prospective study	descriptive statistics
Ciesla et al.[43]	regional	undertriage and overtriage rates	retrospective data analysis	descriptive statistics
Scerbo et al.[77]	single centre	overtriage rate	forecasting model validation	random forest model
Cameron et al.[41]	world-wide	trauma triage approaches and principles	literature review	synthesis of trauma triage literature
Newgard et al.[44]	cross regional	EMS decision-making in trauma triage	mixed-methods study	descriptive statistics and ethnographic surveys
Osen et al.[69]	national	secondary overtriage rate and associated risk factors	retrospective data analysis	descriptive statistics and multiple logistic regression analyses
Ocak et al.[47]	single centre	secondary overtriage rate	retrospective data analysis	descriptive and inferential statistics; logistic regression analyses
Sturms et al.[46]	regional	prehospital triage protocols and triage accuracy (T-RTS Sensitivity)	retrospective data analysis	descriptive statistics
Morris et al.[54]	world-wide	Triage methods for all trauma triage phases	literature review	synthesis of existing literature and quality assessment
Wohlgemut et al.[57]	single centre	triage-based appropriateness of admissions	retrospective, geospatial, observational analysis	descriptive statistics and linear regression analyses
Deeb et al.[59]	regional	undertriage rate and its associated factors	retrospective data analysis	descriptive and inferential statistics; logistic regression analyses
Jammula et al.[53]	regional	undertriage rates and its impact on patient outcome	retrospective data analysis	descriptive statistics and multilevel mixed-effects logistic regression model
Horst et al.[56]	regional	undertriage rate	retrospective data analysis	descriptive statistics and geospatial analysis
Chen et al.[68]	regional	trauma triage criteria and its impact on in-hospital mortality	retrospective data analysis	propensity score matching, mixed-effects logistic regression analyses

Table 5 continued from previous page

Taylor et al.[67]	regional	Helicopter Emergency Medical Services (HEMS) overtriage rate	retrospective data analysis	descriptive statistics and cost analysis
Magnone et al.[187]	single centre	undertriage and overtriage rates	retrospective data analysis	descriptive statistics; univariate and multivariable logistic regression analyses
Sturms et al.[61]	national	undertriage rate and its associated factors	retrospective data analysis	multivariable logistic regression model
Haider et al.[52]	national	undertriage and overtriage rate based on different triage models	triage modelling and retrospective data analysis	descriptive statistics and logistic regression analysis
Garwe et al.[60]	regional	directness of patient transfer to specific trauma centre based on triage	retrospective data analysis	descriptive statistics and propensity score analysis
Curtis et al.[63]	single centre	undertriage rate and its impact on patient outcome	retrospective data analysis	descriptive statistics and logistic regression
Braken et al.[51]	national	undertriage and overtriage rates	retrospective data analysis	descriptive statistics and simulation modeling
Follin et al.[50]	single centre	undertriage and overtriage rates	prospective and observational data analysis	multivariable logistic regression; recursive partitioning based decision trees modeling
Jensen et al.[42]	single centre	undertriage and overtriage rate and their impact on mortality	triage modelling and retrospective data analysis	modern bayesian variable selection techniques; Leave-one-out cross-validated Brier and logarithmic scores for model evaluation
Madiraju et al.[66]	single centre	aeromedical overtriage rates with associated costs and its predictors	retrospective data analysis	descriptive statistics and multivariate logistic regression models
Larsson et al.[78]	cross regional	undertriage and overtriage rates	simulation study based on triage models	descriptive statistics; multivariate logistic regression models; extreme gradient boosting models(XGBoost)
Henriksson et al.[62]	national-wide	undertriage rate,overtriage rate and mistriage rate	predictive modeling study	logistic regression for model development; empirical bootstrapping for model comparison
Shi et al.[75]	cross regional	retriage rate	retrospective data analysis	descriptive statistics and hierarchical logistic regression model
Cohen et al.[76]	single centre	triage decision accuracy in identifying the need for acute care following multi-system trauma	retrospective data analysis	multivariable logistic regression analyses

Table 6. Summary of timeliness studies

References	Research Scope	Evaluation Metrics	Study Design	Analytic Methods
Hewitt et al.[92]	regional	trauma incident timing, frequency and location	retrospective data analysis	descriptive statistics
Dennis et al.[101]	cross-regional	trauma admission volume	forecasting model	artificial neural network (ANN)
Moore et al.[80]	national	hospital LOS and ICU LOS	retrospective data analysis	descriptive statistics and multilevel linear regression analysis
Kieffer et al.[90]	cross-regional	trauma admission timing and frequency	retrospective data analysis	descriptive statistics
Kuimi et al.[87]	regional	trauma admission volume and risk ratio of accessing trauma centres	retrospective data analysis	descriptive statistics and poisson regression methods
Moore et al.[79]	national	hospital LOS and ICU LOS	retrospective data analysis	descriptive statistics and multilevel linear regression analysis
Gomez et al.[91]	regional	timeliness and rates of interfacility transfer, ED LOS	retrospective data analysis	hierarchical regression modeling
Barringer et al.[94]	single centre	hospital LOS, ICU LOS and ventilator days	retrospective data analysis	general linear model
Esposito et al.[93]	regional	interfacility transfer volume	retrospective data analysis	descriptive statistics and logistic regression model
Stonko et al.[100]	single centre	emergent operative cases and daily trauma volume	forecasting model	ANN
Navaratne et al.[88]	single centre	major trauma transfer activity	retrospective data analysis	descriptive statistics
Stonko et al.[102]	single centre	prolonged LOS	forecasting model	ANN
Lin et al.[96]	regional	factors influencing interfacility transfer decisions	qualitative survey	discrete choice modelling (DCM) survey and utility score analysis
Durston et al.[89]	single centre	hospital LOS, ICU LOS and time of patient arrival	retrospective data analysis	descriptive statistics

Prepared using sagej.cls

Table 7. Studies of process indicators and their association with mortality

References	Research Scope	Sample size	research outcome	predictors	research focus	analytics methods
Tiruneh et al. [151]	national	27131	in-hospital mortality	transfer status (direct admissions to trauma centre and interhospital transfer) with controlling confounders(age, gender, population group mechanism, type, ISS, head injuries AIS, SBP, respiratory status, ICU admission, surgery, hospital LOS, evacuation means)	association study	multivariate logistic regression models
Waalwijk et al. [117]	national	1065	24-hour and 30-day mortality	transfer status (direct admissions to lower-level centres vs. transfers to higher-level centres) with controlling confounders (age, gender, dispatch priority, vital parameters, mechanism of injury, ISS, severe injuries, and driving distance to trauma center)	association study	generalized linear models with inverse probability weights
Gallagher et al. [149]	single centre	3415	in-hospital mortality	pretransfer CT scans with controlling confounders (age, sex, race, transport method, ISS, heart rate, intubation status, comorbidity index, GCS, transfer time)	association study	cox regression model with controlling the transfer time
Little et al. [150]	single centre	1424	30-day mortality	day of week of attendance (weekend vs weekday), with controlling for confounders such as age, gender, ISS, GCS, and mechanism of injury	predictors identification	descriptive statistics, Mann-Whitney U test, and odds ratio analysis
Hill et al. [152]	world-wide	22918 (across multiple reviews)	in-hospital mortality, length of stay, and healthcare costs	transfer status (direct admissions vs transfers)	predictor synthesis	systematic review and meta-analysis
Calland and Stukenborg [153]	national	179197	inpatient mortality	trauma centre volume (measured as total cases per facility), with controlling confounders such as age, mechanism of injury, initial systolic blood pressure, initial pulse rate, Glasgow motor score, injury severity score, transfer status, head injury severity, and abdominal injury severity.	association study	Weighted Hierarchical Generalized Linear Models (HGLMs)
Sewalt et al. [154]	national	47157	in-hospital mortality	hospital volume (number of severely injured patients treated per year) with controlling for confounders such as age, sex, ISS, RTS, Charlson Comorbidity Index, penetrating injury, head injury severity, and type of patient referral	association study	meta-analysis and quality assessment
Minei et al. [155]	cross-regional	2070	24-hour mortality and 28-day mortality	trauma centre volume (with increments of 500 patient admissions) with controlling for confounders such as age, gender, pre-hospital systolic blood pressure, Glasgow Coma Scale score, and other relevant clinical and demographic variables	association study	multivariable logistic regression using generalized estimating equations (GEE)

Prepared using sagej.cls

Table 7 continued from previous page

Byrne et al. [156]	national	119740	risk-adjusted ED and overall hospital mortality	prehospital time (90th percentile total prehospital time for each trauma centre) with controlling for confounders such as patient demographics, injury characteristics, and ED physiologic variables	association study	random-intercept multilevel logistic regression models
Berkeveld et al. [157]	single centre	342	in-hospital mortality	prehospital time (with confounders controlled including age, comorbidity based on the ASA-PS score, mechanism of injury, type of injury, HEMS assistance, prehospital Glasgow Coma Scale, and ISS)	association study	binary logistic regression analysis with the use of restricted cubic splines for continuous variables and multivariate adjustment for potential confounders

Prepared using sagej.cls

Table 8. Trauma scoring system

Scoring system	Type of the scoring system	Incorporated variables	Formula mode	Note
Injury Severity Score (ISS)	Anatomical	Abbreviated Injury Scale (AIS) of six classified injured body regions	Sum of the three most severely injured body regions squared AIS range from 0-75. If an injury is classified as having an AIS of 6 (Unsurvivable), the ISS score is automatically set to 75.	<9 = Mild<br>9–15 = Moderate<br>16–24 = Severe<br>≥ 25 = Profound
New Injury Severity Score (NISS)	Anatomical	AIS	The sum of squares of the three most severe injuries AIS, regardless of body region injured	Simplification of the ISS
Revised Trauma Score (RTS)	Physiological	Glasgow coma scale (GCS)<br>Systolic Blood Pressure (SBP)<br>Respiratory Rate (RR)	RTS = α*GCS + α*SBP + α*RESP	The RTS is strongly weighted toward the Glasgow coma scale to account for severe brain injury in the absence of multisystem injury or major physiological abnormalities.
Trauma and Injury Severity Scores (TRISS)	Combination of Anatomical and Physiological	ISS, RTS, and age index	Ps = 1/(1 + e-b)<br>Where ‘b’ is calculated from:<br>b = b0 + b1(RTS) + b2(ISS) + b3(Age Index)	The TRISS calculator determines the probability of survival
